# The influence of the environment and indoor residual spraying on malaria risk in a cohort of children in Uganda

**DOI:** 10.1038/s41598-022-15654-0

**Published:** 2022-07-07

**Authors:** Margaux L. Sadoine, Audrey Smargiassi, Ying Liu, Philippe Gachon, Guillaume Dueymes, Grant Dorsey, Michel Fournier, Joaniter I. Nankabirwa, John Rek, Kate Zinszer

**Affiliations:** 1 School of Public Health, Université de Montréal, Montréal, Québec Canada; 2 Public Health Research Center, Université de Montréal, Montréal, Québec Canada; 3 ESCER (Étude et Simulation du Climat à l’Échelle Régionale) Centre, Université du Québec à Montréal, Montréal, Québec Canada; 4 University of California San Francisco, San Francisco, USA; 5 Montreal Regional Department of Public Health, Montréal, Québec Canada; 6 Infectious Disease Research Collaboration, Kampala, Uganda; 7 Department of Medicine, Makerere University College of Health Sciences, Kampala, Uganda

**Keywords:** Malaria, Environmental impact

## Abstract

Studies have estimated the impact of the environment on malaria incidence although few have explored the differential impact due to malaria control interventions. Therefore, the objective of the study was to evaluate the effect of indoor residual spraying (IRS) on the relationship between malaria and environment (i.e. rainfall, temperatures, humidity, and vegetation) using data from a dynamic cohort of children from three sub-counties in Uganda. Environmental variables were extracted from remote sensing sources and averaged over different time periods. General linear mixed models were constructed for each sub-counties based on a log-binomial distribution. The influence of IRS was analysed by comparing marginal effects of environment in models adjusted and unadjusted for IRS. Great regional variability in the shape (linear and non-linear), direction, and magnitude of environmental associations with malaria risk were observed between sub-counties. IRS was significantly associated with malaria risk reduction (risk ratios vary from RR = 0.03, CI 95% [0.03–0.08] to RR = 0.35, CI95% [0.28–0.42]). Model adjustment for this intervention changed the magnitude and/or direction of environment-malaria associations, suggesting an interaction effect. This study evaluated the potential influence of IRS in the malaria-environment association and highlighted the necessity to control for interventions when they are performed to properly estimate the environmental influence on malaria. Local models are more informative to guide intervention program compared to national models.

## Introduction

Uganda is one of six countries accounting for half of the global malaria cases, with more than 14 million cases confirmed in 2020^1^. The disease is endemic in 95% of the country and accounts for a significant portion of the disease burden with 27–34% of outpatient visits and 19–30% of hospitalizations due to malaria^2^. Malaria control in Uganda is oriented in indoor residual spraying (IRS) program targeting epidemic-prone areas since 2006, and nationwide campaigns have been conducted in 2013–2014 and 2017–2018, aiming to achieve universal insecticide-treated nets (ITN) coverage. By 2018–2019, 83% of households owned at least one ITN and 74% of households in districts targeted by indoor residual spraying measures had received the intervention^3^. Following the sustained malaria control efforts, there has been evidence of a significant decrease in malaria burden over the last decade, with a reduction of nearly 1.5 million cases between 2017 and 2018 ^4^.

While the main determinants of malaria risk are known, there is a large temporal and spatial (intra- and inter-regional) variability in the influence of these factors that requires in-depth analyses at sub-national scales to orient interventions. Indeed, patterns of association between malaria and weather rely on geographic and climatic context^5^, as the effectiveness of vector control interventions^6,7^. A few studies have demonstrated that adherence to certain interventions depends on environmental conditions, for example, high temperatures negatively influence the use of long-lasting insecticidal nets (LLINs)^8,9^, while increase rainfall positively influence net use through perceived malaria risk associated to mosquito abundance^10^.

Additionally, it is possible that control interventions may impact the influence of the environment on malaria, which has been highlighted in only two studies^11,12^. Chaves and al.^11^ showed a reduction of between 30 to 80% of the average effect of temperatures on malaria prevalence for *P. falciparum* and *P. vivax* following a mass distribution campaign of ITN, while Carrasco-Escobar and al.^12^ showed a time-varying change in slope in the dose–response association between environmental factors and malaria prevalence after community interventions.

The analysis of the effect of interaction between environment and interventions, as well as their joint effect on malaria, are therefore, often not considered in the literature^13^.

The majority of studies that have analyzed meteorological factors and malaria risk averaged the environmental and/or meteorological measures over a one week or one month period, with or without lags. Lags or delays are typically investigated up to 12 weeks for weekly data and up to 12 months for monthly data and most studies showed a lag period of between 7 to 12 weeks for temperature and 8 to 12 weeks for precipitation^14^. However, the use of weekly or monthly lagged environmental covariates does not allow for the timeframe necessary to account for the cumulative effects of environmental covariates^15^. Malaria symptom onset is a result of several processes including the developmental period of the mosquito and parasite within the mosquito, and the incubation period of parasites within the human body. Averaging climatic factors over several months may be necessary to account for these biological mechanisms and has been used to analyse mosquito density^16–18^, but rarely for symptomatic cases of malaria^19,20^.

An often important and overlooked aspect of environmental and meteorological measures and malaria risk is non-linearity. There are optimal and sub-optimal temperature, precipitation, and humidity thresholds for the development of mosquitoes and parasites^21,22^. Not considering the non-linear trend of malaria risk factor can result in biased estimates of association, leading to inaccurate projections^5^.

Therefore, in this study, the joint effects of IRS and different environmental factors averaged over different time periods were examined in a cohort of Ugandan children. The nonlinear relationships between environmental factors and malaria incidence were explored and comparisons between the average periods of 20 days to 120 days and the weekly and bi-weekly averages lagged up to 16 weeks were made. A comparison between the results of a pooled model and regional models was also conducted.

## Results

### Descriptive results

A total of 1090 children from 331 total households were followed between July 2011 and July 2017 (Table 1). The mean duration of follow-up was 3.3 years, with an average of 35 total visits and 5 malaria episodes per child. The proportion of malaria episodes represented 17% of the total visits. Households had an average of 6.55 individuals and 540 participants (49.5%) were female.

**Table 1:** Characteristics of study participants and households.

	Kihihi (N = 377)	Nagongera (N = 375)	Walukuba (N = 338)	Total (N = 1090)
**Sex**				
Female	199 (52.8%)	174 (46.4%)	167 (49.4%)	540 (49.5%)
Male	178 (47.2%)	201 (53.6%)	171 (50.6%)	550 (50.5%)
**Age at enrollment**				
Mean ± SD	4.54 ± 2.81	4.16 ± 2.74	3.97 ± 2.65	4.23 ± 2.74
Min–Max	0.500–9.87	0.500–9.98	0.480–9.96	0.480–9.98
**Household wealth index**				
Poorest	122 (32.4%)	176 (46.9%)	95.0 (28.1%)	393 (36.1%)
Middle	128 (34.0%)	124 (33.1%)	87.0 (25.7%)	339 (31.1%)
Least poor	127 (33.7%)	75.0 (20.0%)	156 (46.2%)	358 (32.8%)
**Household size**				
Mean ± SD	6.51 ± 2.29	6.95 ± 3.44	6.15 ± 2.96	6.55 ± 2.95
Min–Max	2.00–12.0	2.00–23.0	2.00–17.0	2.00–23.0
**Dwelling type**				
Modern	95.0 (25.2%)	64.0 (17.1%)	158 (46.7%)	317 (29.1%)
Traditional	282 (74.8%)	311 (82.9%)	180 (53.3%)	773 (70.9%)
**Number of visits during follow-up**				
Mean ± SD	31.0 ± 17.2	48.2 ± 29.4	24.8 ± 14.8	35.0 ± 23.8
Min–Max	2.00–83.0	3.00–130	2.00–67.0	2.00–130
**Number of malaria episodes**				
Mean ± SD	6.44 ± 7.64	7.82 ± 7.50	0.837 ± 1.49	5.18 ± 7.00
Min–Max	0–43.0	0–35.0	0–14.0	0–43.0
**Follow-up duration (year)**				
Mean ± SD	3.38 ± 1.46	3.73 ± 1.75	2.78 ± 1.57	3.31 ± 1.65
Min–Max	0.0384–4.86	0.142–5.86	0.104–4.88	0.0384–5.86

Nagongera experienced more precipitation with an average of 86 mm of cumulative precipitation over 20 days and up to 519 mm over 120 days, compared to 73 mm and 436 mm over 20 days and 120 days respectively in Kihihi; 77.5 mm and 458 mm over 20 days and 120 days respectively in Walukuba (Supplementary Table S2). Walukuba had higher minimum temperatures (20-day average: 19.5˚C, range: 17.9–21.7˚C), while maximum temperatures were higher in Nagongera (20-day average: 29˚ C, range: 26.7–33.9˚C) (Supplementary Table S2).

For LLINs, self reported use was > 99% in every sub-county during follow up for all three sites (Supplementary Fig. S1). LLIN use was therefore not considered in the models as there was no variation in the response. The proportions of households sprayed in Nagongera were 96.9%, 95.6%, 96.8% proportion of households sprayed respectively (data not available for the phase 4).

### Bivariate analysis

Analysis of the bivariate relationships between malaria and meteorological variables for the clustered sub-counties showed nonlinear relationships for cumulative precipitation and humidity averaged between 20 and 120 days. Nonlinear trends were also observed for maximum temperatures, most apparent at a 120-days average (Supplementary Fig. S2). Analysis by subcounty showed greater variations between subcounties and between different averaging periods for the same subcounty (Figs. S3–S5).

### Multivariable analysis for pooled and sub-counties models

Pooled models with meteorological variables averaged over 1 or 2 weeks, lag up to 16 weeks, showed higher AIC than pooled models with unlagged environmental variables averaged between 20 and 120 days (Supplementary Tables S3–S5). The lowest AIC for lagged environmental variables was obtained at 13 weeks for a 7-days average and 16 weeks for a 14-days average. However, among all pooled multivariate models (lagged and unlagged environmental variables), the smallest AIC was obtained for the meteorological variables unlagged, averaged over a 90-days period . For models at each subcounty, the smallest AIC was obtained for the meteorological variables averaged over a 120-days period for Walukuba, over a 90-days period for Nagongera, and over a 20-days period for Kihihi (Supplementary Table S3).

**Figure 1:**
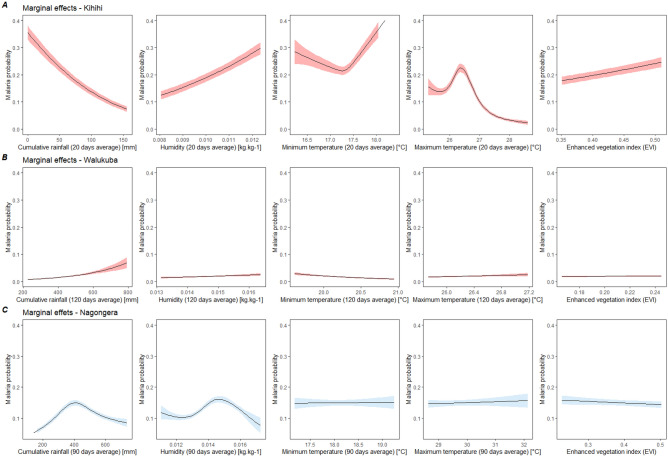
Marginal effects of environmental variables on malaria risk from GLMM models for each subcounty: Kihihi (**A**), Walukuba (**B**) and Nagongera (**C**). Models are adjusted for maximum and minimum temperatures, rainfall, humidity, EVI, age at visit, sex, housing type, household wealth index, number of persons living in the house, number of meat meal in all three models and Nagongera model is additionally adjusted for IRS. Models in red are not adjusted for intervention (IRS); models in blue are adjusted for IRS.

Comparing the marginal effects of environmental variables on malaria at each subcounty, large variations in the direction and shape of the relationships were observed between regions (Fig. 1). Linear trends were observed for all environmental variables in Walukuba, for rainfall, humidity and EVI in Kihihi, for maximum and minimum temperatures and EVI in Nagongera. Coefficients of environmental variables with linear trends are presented in Tables 2, 3, 4 and 5. Malaria risks significantly increased with the increase in rainfall in Walukuba (RR = 13.59, 95% CI [4.25–43.50]) with the increase in EVI (RR = 12.80, 95% CI [4.71–34.81]) and humidity in Kihihi (RR = 5.36, 95% CI [3.31–8.67]) (Tables 3 and 4). An increase in minimum temperatures and rainfall were significantly associated with risk reduction in Walukuba and Kihihi, respectively (RR = 0.13, 95% CI [0.02–0.67]; RR = 0.08, 95% CI [0.05–0.12]).

**Table 2:** Summary of GLMM models for Nagongera.

Predictors	Nagongera (without IRS)			Nagongera (with IRS)		
	Risk ratios	CI (95%)	*P*-Value	Risk ratios	CI (95%)	*P*-Value
Intercept	0.01	0.00 – 0.02	** < 0.001**	0.08	0.04 – 0.18	** < 0.001**
Sex [Male]	1.18	1.04 – 1.35	**0.011**	1.16	1.01 – 1.32	**0.031**
Age at visit (years)	0.93	0.91 – 0.95	** < 0.001**	0.96	0.94 – 0.99	**0.001**
Dwelling type [Traditional]	1.10	0.87 – 1.39	0.431	1.11	0.88 – 1.41	0.374
Meal with meat per week	0.98	0.93 – 1.02	0.308	0.98	0.93 – 1.03	0.433
Household wealth index [Middle]	1.24	0.96 – 1.59	0.099	1.22	0.94 – 1.57	0.132
Household wealth index [Poorest]	1.16	0.91 – 1.48	0.236	1.16	0.90 – 1.48	0.251
Number of persons living in house	1.02	0.99 – 1.05	0.128	1.02	0.99 – 1.05	0.141
Enhance vegetation index	15.99	7.10 – 36.01	** < 0.001**	0.66	0.25 – 1.72	0.395
Minimum temperatures (90 days) (˚C)	0.01*	0.01 – 0.02	** < 0.001**	1.03*	0.49 – 2.19	0.936
Maximum temperatures (90 days) (˚C)	1.31*	0.81 – 2.12	0.275	1.12*	0.61 – 2.03	0.721
IRS [Phase 1]				0.35	0.28 – 0.42	** < 0.001**
IRS [Phase 2]				0.19	0.15 – 0.24	** < 0.001**
IRS [Phase 3]				0.05	0.03 – 0.08	** < 0.001**
IRS [Phase 4]				0.09	0.06 – 0.12	** < 0.001**
IRS [Phase 5]				0.21	0.15 – 0.30	** < 0.001**
**Random effects**						
σ^2^	3.29			3.29		
τ_00_	0.14 _Participants_			0.15 _Participants_		
	0.06 _Households_			0.06 _Households_		
ICC	0.04 _Participants_			0.04 _Participants_		
	0.01 _Households_			0.01 _Households_		
N	375 _Participants_			375 _Participants_		
	107 _Households_			107 _Households_		
Observations	18,071			18,071		
Marginal R^2^/Conditional R^2^	0.236/0.281			0.333 / 0.374		

Significant values are in bold.

*Risk ratio of malaria when the meteorological linear variable changes from its minimum to its maximum value.

**Table 3:** Summary of GLMM model for Walukuba.

Predictors	Walukuba		
	Risk ratios	CI (95%)	*P*-Value
Intercept	0.01	0.00 – 0.06	** < 0.001**
Sex [Male]	1.01	0.73 – 1.40	0.929
Age at visit (years)	0.98	0.92 – 1.04	0.515
Dwelling type [Traditional]	0.91	0.51 – 1.65	0.769
Meal with meat per week	1.03	0.90 – 1.18	0.658
Household wealth index [Middle]	1.18	0.62 – 2.26	0.618
Household wealth index [Poorest]	1.91	0.93 – 3.95	0.079
Number of persons living in house	0.95	0.87 – 1.04	0.247
Enhance vegetation index	2.89	0.01 – 667.58	0.702
Minimum temperatures (120 days) (˚C)	0.13*	0.02 – 0.67	**0.015**
Maximum temperatures (120 days) (˚C)	2.25*	0.45 – 11.32	0.327
Cumulative rainfall (120 days) (mm)	13.59*	4.25 – 43.50	** < 0.001**
Humidity (120 days) (kg.kg^-1^)	2.53*	0.72 – 8.93	0.149
**Random effects**			
σ^2^	3.29		
τ_00_ _Participants_	0.18		
τ_00_ _Households_	0.84		
ICC _Participants_	0.05		
ICC _Households_	0.20		
N _Participants_	338		
N _Households_	117		
Observations	8355		
Marginal R^2^/Conditional R^2^	0.069/0.289		

Significant values are in bold.

*Risk ratio of malaria when the meteorological linear variable changes from its minimum to its maximum value.

**Table 4:** Summary of GLMM model for Kihihi.

Predictors	Kihihi		
	Risk ratios	CI (95%)	*P*-Value
Intercept	0.03	0.01 – 0.07	** < 0.001**
Sex [Male]	1.13	0.96 – 1.32	0.157
Age at visit (years)	1.01	0.98 – 1.03	0.655
Dwelling type [Traditional]	1.61	1.09 – 2.38	**0.018**
Meal with meat per week	0.84	0.71 – 1.00	0.053
Household wealth index [Middle]	1.73	1.17 – 2.56	**0.006**
Household wealth index [Poorest]	1.88	1.23 – 2.87	**0.004**
Number of persons living in house	1.07	1.00 – 1.15	0.054
Enhance vegetation index	12.80	4.71 – 34.81	** < 0.001**
Cumulative rainfall (20 days) (mm)	0.08*	0.05 – 0.12	** < 0.001**
Humidity (20 days) (kg.kg^-1^)	5.36*	3.31 – 8.67	** < 0.001**
**Random effects**			
σ^2^	3.29		
τ_00_ _Participants_	0.19		
τ_00_ _Households_	0.47		
ICC _Participants_	0.05		
ICC _Households_	0.12		
N _Participants_	377		
N _Households_	107		
Observations	11,667		
Marginal R^2^/Conditional R^2^	0.120/0.267		

Significant values are in bold.

*Risk ratio of malaria when the meteorological linear variable changes from its minimum to its maximum value.

**Table 5:** Summary of pooled GLMM model with and without IRS.

Predictors	Global model (without IRS)			Global model (with IRS)		
	Risk ratios	CI (95%)	*P*-Value	Risk ratios	CI (95%)	*P*-Value
Intercept	0.02	0.01 – 0.03	** < 0.001**	0.04	0.02 – 0.07	** < 0.001**
Sex [male]	1.15	1.04 – 1.27	**0.007**	1.13	1.02 – 1.25	**0.020**
Age at visit (years)	0.97	0.96 – 0.99	** < 0.001**	0.99	0.97 – 1.01	0.194
Dwelling type [Traditional]	1.28	1.03 – 1.60	**0.026**	1.28	1.02 – 1.59	**0.029**
Meal with meat per week	0.96	0.90 – 1.01	0.134	0.96	0.90 – 1.01	0.135
Household wealth index [Middle]	1.46	1.16 – 1.84	**0.001**	1.42	1.13 – 1.80	**0.003**
Household wealth index [Poorest]	1.53	1.19 – 1.96	**0.001**	1.53	1.19 – 1.97	**0.001**
Number of persons living in house	1.02	0.99 – 1.06	0.223	1.02	0.99 – 1.06	0.252
Enhance vegetation index	15.76	8.68 – 28.61	** < 0.001**	3.26	1.69 – 6.31	** < 0.001**
Minimum temperatures (90 days) (˚C)	0.08*	0.06 – 0.12	** < 0.001**	2.38*	1.51 – 3.76	** < 0.001**
Maximum temperatures (90 days) (˚C)	0.32*	0.22 – 0.46	** < 0.001**	0.28*	0.18 – 0.42	** < 0.001**
Subcounty [Nagongera]	1.10	0.86 – 1.40	0.459	1.72	1.34 – 2.21	** < 0.001**
Subcounty [Walukuba]	0.34	0.25 – 0.47	** < 0.001**	0.11	0.08 – 0.15	** < 0.001**
IRS [Phase 1]				0.45	0.38 – 0.55	** < 0.001**
IRS [Phase 2]				0.17	0.13 – 0.21	** < 0.001**
IRS [Phase 3]				0.03	0.02 – 0.05	** < 0.001**
IRS [Phase 4]				0.08	0.06 – 0.11	** < 0.001**
IRS [Phase 5]				0.26	0.19 – 0.34	** < 0.001**
**Random effects**						
σ^2^	3.29			3.29		
τ_00_	0.18 _Participants_			0.18 _Participants_		
	0.37 _Households_			0.37 _Households_		
ICC	0.05			0.05 _Participants_		
	0.10			0.10 _Households_		
N	1090 _Participants_			1090 _Participants_		
	331 _Households_			331 _Households_		
Observations	38,093			38,093		
Marginal R^2^/Conditional R^2^	0.230/0.340			0.282/0.385		

Significant values are in bold.

*Risk ratio of malaria when the meteorological linear variable changes from its minimum to its maximum value.

Non-linear relationships were observed for precipitation and humidity in Nagongera (Fig. 1). Percentage change in risk between percentiles for nonlinear variable are presented in Table 6. As an example, an increase from 246 to 355 mm in cumulative rainfall over 90 days resulted in a 48.1% increase in malaria risk, and a 57% decrease in risk between 457.5 and 737 mm (Table 6). Minimum and maximum temperatures at Kihihi also exhibited nonlinear trends (Fig. 1 and Table 6). The magnitudes of environmental influences were higher in Kihihi compared to other sub-counties and lowest in Walukuba (Fig. 1).

**Table 6:** Percentage change in risk between the 25th and the 50th, the 50th and the 100th percentile for nonlinear predictors.

	Q1*	Q2*	Q4*	Risk at Q1	Risk at Q2	Risk at Q4	Difference in risk between Q1 and Q2 (%)	Difference in risk between Q2 and Q4 (%)
**Pooled model (without IRS)**								
Cumulative rainfall	246.1	355.2	737.0	0.079	0.117	0.043	48.1	− 63.2
Humidity	0.0122	0.0132	0.0172	0.087	0.121	0.031	39.1	− 74.4
**Pooled model (with IRS)**								
Cumulative rainfall	246.1	355.2	737.0	0.072	0.100	0.045	38.9	− 55.0
Humidity	0.0122	0.0132	0.0172	0.087	0.102	0.077	17.2	− 24.5
**Nagongera model (without IRS)**								
Cumulative rainfall	246.1	355.23	737.0	0.101	0.180	0.078	78.2	− 56.7
Humidity	0.0122	0.0132	0.0172	0.144	0.194	0.021	34.7	− 89.2
**Nagongera model (with IRS)**								
Cumulative rainfall	246.1	355.23	737.0	0.099	0.149	0.084	50.5	− 43.6
Humidity	0.0122	0.0132	0.0172	0.112	0.153	0.076	36.6	− 50.3
**Kihihi model**								
Minimum temperature	17.2	17.3	18.4	0.219	0.215	0.447	− 1.8	107.9
Maximum temperature	25.9	26.3	28.6	0.158	0.222	0.024	40.5	-89.2

* Units are **˚**C for temperatures, kg.kg^-1^ for humidity and mm for rainfall.

**Figure 2:**
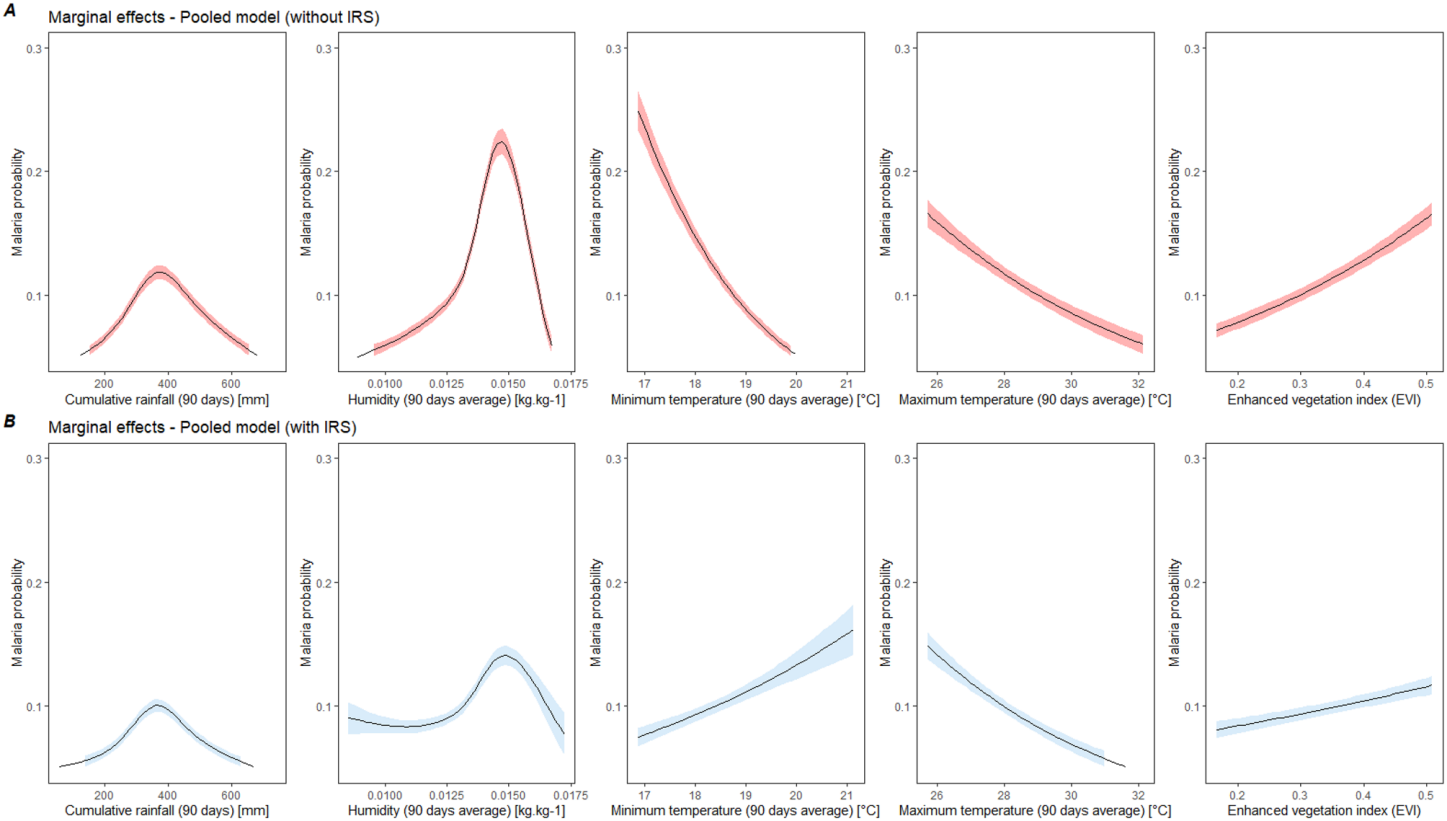
Marginal effect of environmental variables on malaria risk from global GLMM model not controlling for IRS (**A**) and controlling for IRS (**B**). Models without interventions include maximum and minimum temperature, rainfall, humidity, EVI, age at visit, sex, housing type, household wealth index, number of persons living in the house, number of meat meal. Models with intervention additionally include the spraying variable.

**Figure 3:**
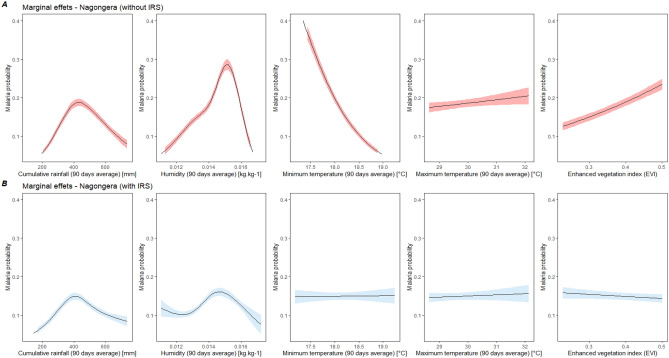
Marginal effect of environmental variables on malaria risk from Nagongera model not controlling for IRS (**A**) and controlling for IRS (**B**). Models without interventions include maximum and minimum temperature, rainfall, humidity, EVI, age at visit, sex, housing type, household wealth index, number of persons living in the house, number of meat meal. Models with intervention additionally include the spraying variable.

Comparison of the marginal effects of meteorological variables for the pooled model with and without IRS showed a reduction in the magnitude of the effect of all variables when considering IRS, with a greater reduction for humidity (− 37.2%) and EVI (− 29.6%) (Fig. 2 and Supplementary Table S6). The influence of minimum temperature changed direction in the model controlling for IRS, compared to the model without IRS. The same comparison was done for Nagongera (the only sub-county to have received IRS during the study period) and results showed a change in the direction of the influence of minimum temperatures and vegetation on malaria (Fig. 3). In Nagongera model not controlling for IRS, minimum temperatures significantly decreased the risk of malaria (RR = 0.01, 95% CI [0.01–0.02]) and EVI significantly increased the risk of malaria (RR = 15.99, 95% CI [7.10–36.01]). When controlling for IRS, these associations were in opposite directions (increased risk associated with an increase in minimum temperature and decreased risk associated with an increase in EVI) and they also lost their statistical significance (Fig. 3 and Table 2). The maximum effect of humidity, rainfall, and maximum temperature were reduced by 43.8%, 20.2% and 23.6%, respectively, when controlling for IRS (Supplementary Table S6).

Regarding interventions in Nagongera, each round of spraying was significantly associated with risk reduction (risk ratios vary from RR = 0.03, CI 95% [0.03–0.08] to RR = 0.35, CI 95% [0.28–0.42]), with the biggest reduction observed during the third round of spraying (Table 2). Phase 5 was marked by a slight increase in risk (Table 2).

Results of the models’ diagnosis are presented in Supplementary Figs. S10–S13. Diagnosis showed no evidence of overdispersion in the residuals of the pooled and individual sub-county models.

## Discussion

The results demonstrated that there is important regional variability in the shape (linear and non-linear), direction, and magnitude of environmental associations with malaria risk in Uganda. The results have also shown that in the context of a stable and perennial transmission setting, IRS was effective in reducing malaria risk and adjusting models for IRS modifies the magnitude of environmental effects.

Malaria transmission is characterized by complex and sometimes non-linear relationships with its determinants. The development and survival of mosquitoes depend on optimal thresholds of temperatures and precipitation^23,24^, which has been shown both experimentally and epidemiologically, and underscores the importance of considering these thresholds. Historically, studies have tended to overlook this aspect, although there has been an increase in the use of distributed lag non-linear models (DLNMs)^25^. Exploring non-linear associations between the environment and malaria can reveal important information about malaria epidemiology and ultimately, aid in planning and optimizing control measures. For example, IRS has been demonstrated to be more effective if conducted early in the transmission cycle and during high transmission years^7^, which could suggest that the effectiveness of interventions may depend on the level of intensity of environmental variables. Specifically, high humidity levels have been shown to play a major role in the long-term residual effectiveness of indoor spraying^26^. Other studies have demonstrated that temperatures variations can affect both mosquito physiology^27,28^ and the toxicity of chemicals used for malaria control (i.e. IRS, ITNs and LLINs)^29,30^.

The variations in environmental associations with malaria are often observed between regions, due to differing contexts as well as methodological differences between studies^5,14,31^. The results of the present study have shown that the time periods for considering the influence of environmental variables vary by region, such as the magnitude and the direction of environmental effects.

The need for local models has been demonstrated by other studies^32–34^. Risk factors not only differ between regions but also at a smaller scale, on relatively short distances^34^. Local conditions are the main drivers of malaria transmission^35^ and include, among others, vector population characteristics, biophysical environment, and seasonality^32^. High-resolution analysis of local variations of malaria risk in endemic and epidemic regions may be more informative to guide control programs.

Lagged environmental variables did not necessarily provide better statistical models in terms of model fit compared to averaging periods. This may suggest that long averaging periods could be suitable to capture both the different stages of the transmission cycle and the environmental mechanisms that influence the different stages. For example, the influence of precipitation may need to be analyzed over long periods given the time required for water to accumulate in breeding sites and its influence that can persist for up to several weeks^14^. Some studies have investigated the effect of longer lag lengths to analyze exposure effects over time^31^ and found up to 20–28 weeks lag of the association between malaria and precipitation ^14^. In our analysis, long averaging periods for environmental effects were identified for Walukuba and Nagongera (120 days and 90 days, respectively). Other studies^20,36^ in Uganda have reported significant associations for long periods such as precipitation averaged over 4 months, as well as surface temperatures averaged over 2-months with variable temporal associations according to transmission setting.

Findings from this study also have shown the effectiveness of IRS, which has been supported by several other studies^37–43^, and recent work by our group demonstrated the critical role of continuous spraying in achieving global malaria targets^44^. Interestingly, when comparing pooled models not controlling and controlling for IRS, the influence of all environmental variables on malaria risk were attenuated in the latter. For the Nagongera model with IRS, the influence of minimum temperature and EVI were in the opposite direction to those of the Nagongera model without IRS, while the other variables (rainfall, humidity, maximum temperatures) were attenuated. The difference in estimates between models with and without IRS indicates a certain level of residual confounding, and the association between environment and malaria would be inaccurate or overestimated if IRS was not properly captured. It could also suggest an interaction effect between environment and interventions. This would imply that the influence of the environment on malaria risk could be modulated by the IRS and/or that the influence of the IRS varies according to the intensity of the environmental variables. Disregarding the interaction of two variables would lead to an incomplete conclusion of their influence, as it would be solely based on their main effects. Therefore, the results of our study should be interpreted with caution.

This study has several limitations. First, the influence of weather was studied using the same period for averaging each meteorological variable in each model. This type of assumption limits the consideration of the different biological mechanisms as the time between the onset of the disease and the weather varies according to the type of meteorological variable^45^. For example, longer times periods can be expected for precipitation than for temperatures due to the time required for rainwater to accumulate and supply breeding sites^46^. Fixed lags also limit the plausibility of associations at the population level and may lead to inconsistent results between study sites^47^. Analysing the patterns of associations individually between environmental variables and malaria would be an improvement to correct for potential biases. Secondly, the independent impact of LLINs could not be estimated, as all participants reported sleeping under a mosquito net throughout the study period. Self-report measurement of net use is often biased in favour of greater use than actual use and a very recent study from this cohort showed that non-adherence was high among both children (< 5 years) and school-aged children (5–17 years) when adherence was assessed at the home rather than at the clinic^9^. This implies that the variation in use was not properly captured and that LLIN may represent an important residual confounder^48,49^. Having data on LLIN hung inside houses could be used as a proxy of LLIN usage, but they are rarely available due to the difficulty of collecting such information. Finally, although the data are from various transmission settings, they are limited to three regions and a young population, which limits the generalizability of the results. Using data from several regions would provide a better picture of the characteristics that distinguish the regions or make them comparable.

## Conclusion

Significant progress has been made in the fight against malaria and improved knowledge of the determinants of the infection and their dynamics have been key to malaria control progress. This study is one of the few to have considered the joint effects of vector interventions and multiple environmental factors, as well as the potential influence of IRS on the malaria-environment relationship. Results showed a great variability in the environment-malaria association according to various transmission settings. Indoor residual spraying was effective in reducing the burden of malaria in high transmission intensity areas and model adjustment for this intervention changed the magnitude or direction of environmental associations with malaria. Therefore, when interventions against malaria have been conducted, it is important to appropriately consider it in statistical analyses to avoid incorrect estimates of the environmental influence. Further research should explore the possibility of using averaging periods with distinct pattern for each environmental variables instead of lags and consider building local models versus national models.

## Methods

### Study site and population

This study is based on data collected from a prospective cohort of children from three sub-counties in Uganda, that were chosen to represent various transmission settings. Walukuba is a peri-urban area in South Central Uganda with a low malaria transmission intensity; Kihihi is a predominantly rural area in southwestern Uganda with moderate malaria transmission; and Nagongera is a predominantly rural area in southeastern Uganda with very high transmission intensity^50^. Transmission in all these areas is perennial, with two annual peaks following the rainy seasons (March to May and August to October).

Details on participants selection have been described elsewhere^50^. Briefly, all eligible children aged 0.5–10 y and their primary caregiver were enrolled in August–September 2011 from 300 households (100 per site) randomly selected from enumeration surveys conducted in the three sub-counties. Recruitment was dynamic such that all newly eligible children were enrolled during follow-up. Children from 31 randomly selected additional households were enrolled between August and October 2013 to replace households in which all study participants had been withdrawn and were followed using the same procedures described above.

Written informed consent was provided by parents/guardians. At enrolment, study participants were given a long-lasting insecticide net (LLIN) and underwent a standardized evaluation including a history, physical examination, and collection of blood for hemoglobin. Cohort participants received no incentives to participate other than receiving all medical care free of charge at designated study clinics open every day and were reimbursed for clinic travel expenses. Participants were invited for routine visits to the study clinic every 3 months, with the frequency of routine visits increasing to every 30 days from December 2014. Parents or guardians were encouraged to bring their children to the clinic any time they were ill and participants who required inpatient care were referred to the local district hospital. At each of these visits, blood was obtained by pricking the finger for a thick blood smear. Episodes of malaria were diagnosed by the detection of asexual parasites via microscopy with the presence of fever within the past 24 h or an elevated temperature (≥ 38.0 ℃ tympanic). Study participants were withdrawn from the study for (a) permanent move out of the sub-county, (b) inability to be located for > 4 months, (c) withdrawal of informed consent, (d) inability to comply with the study schedule and procedures, or (e) reaching 11 y of age. Flow diagram of participants included in the analyses can be found in ClinEpiDB repository at https://clinepidb.org/ce/app/record/dataset/DS_0ad509829e .

### Malaria control interventions

Every study participant was provided a LLIN at enrolment. LLIN use was captured during each clinic visit and defined as whether the participant reported sleeping under an LLIN the previous night.

Indoor residual spraying (IRS) program has been carried out in high-transmission areas since 2006. Concerning our study sites, Walukuba sub-county did not receive IRS and Kihihi sub-county received a single round of IRS using the pyrethroid lambda-cyhalothrin in February–March 2007, over 4 years before the launch of the cohort, which was therefore not considered in our analysis. IRS was introduced for the first time in Nagongera sub-county in December 2014. Four rounds of spraying were delivered during the study period: December 2014–February 2015 (phase 1), June–July 2015 (phase 2), November–December 2015 (phase 3) and June–July 2016 (phase 4). The first three rounds were administered using the carbamate bendiocarb, and the last one using the organophosphate Actellic CS. As determined by a previous study^37^ significant resurgences of incidence began at week 24 for IRS 1, week 32 for IRS 3, and week 44 for IRS 4, but none was detected after round 2. This information was used in this current study to create a factor type variable, using the start date of each spraying round as the starting point of each phase: no spraying, phase 1 (from IRS round 1 starting date to 24 weeks after), phase 2 (from IRS round 2 to IRS round 3), phase 3 (from IRS round 3 to 32 weeks after), phase 4 (from IRS round 4 to 44 weeks after), phase 5 (beyond the changepoint of round 4). The “no spraying” phase was defined as the reference for statistical analysis.

### Environmental data

Environmental variables were extracted from remote sensing sources. Daily precipitation (mm/day) for Uganda during 1989–2020 at 0.1° spatial resolution were obtained from The Africa Rainfall Climatology Version 2 (ARC2)^51^. Daily maximum and minimum temperature datasets at 0.25° × 0.25° spatial resolution from years 1979–2018 and the hourly near-surface specific humidity dataset for Uganda during 1979–2018 at 0.5° × 0.5° spatial resolution were obtained from reanalysis product—the European Centre for Medium-Range Weather Forecasts (ECMWF) Reanalysis 5 (ERA5). These datasets were produced by applying the WATCH Forcing Data methodology to the surface meteorological variables from the ERA5 reanalysis, and correspond to bias-corrected reconstruction of near-surface data downgraded at a resolution of around 0.5°^52^. The gridded hourly datasets were further aggregated into daily averages.

The 16-day Enhanced Vegetation Index (EVI) dataset with a spatial resolution of 0.05 × 0.05° for Uganda was extracted from the Moderate Resolution Imaging Spectroradiometer vegetation indices products (MOD13A1)^53^. This dataset was available from the U.S. Geological Survey (USGS: https://modis.gsfc.nasa.gov/data/). The EVI imagery products collected between January 1 to January 16 and June 10 to June 26 of each year from 2010 to and 2020 were downloaded to represent the vegetation coverage for the dry and rainy seasons, respectively.

Daily weather variables data were produced for the three sub-counties of interest by averaging data from parishes within the sub-counties; there were 3, 14 and 7 parishes in Walukuba, Kihihi, and Nagongera, respectively. Daily temperatures (mean, minimum and maximum) and humidity were then averaged over 20, 30, 60, 90 and 120 days prior to each clinic visit of each individual. Cumulative rainfall (mm) for the same time periods were created. Two other averaging periods for these variables were also produced for 7 and 14 days and lagged by one week up to 16 weeks, to perform sensitivity analyzes by comparing lags to averaging periods.

### Statistical analysis

The first step of analysing the association between environmental covariates, IRS, and the risk of malaria positivity during multiple visits of children consisted in investigating the shape of the relationship between meteorological variables averaged for each period and malaria risk, through bivariate analysis. Non-linear relationships were subsequently considered in multivariable models using natural cubic splines. Spearman correlation analysis was conducted to examine correlation between weather variables. Mean temperatures presented relatively high correlations with maximum temperatures (> 0.7) and minimum temperatures (> 0.5). It was therefore chosen to only consider maximum and minimum temperatures in the subsequent analyzes.

Generalized linear mixed models (GLMMs) were created to analyze the risk of malaria in the three sub-counties combined (pooled model) and in each sub-county separately. The models were based on a log-binomial distribution and accounted for repeated measures and clustering by household with random effects. Multivariable models included the environmental variables (maximum and minimum temperatures, rainfall, humidity, and EVI) with the same averaging period or lag for each of the environmental variables for a specific model, vector control interventions (IRS—no spraying, phase 1 to 5), age at visit, sex, housing type (traditional vs modern—traditional houses are characterized by thatched roofs, mud walls and open eaves while, modern houses have metal roofs, brick or concrete walls and closed eaves), household wealth index (poorer, middle, less poor—the wealth index was developed in a previous study ^54^ based on principal component analysis of various households assets of this population), and the number of persons living in the house. The number of meat meals per week was also included as a previous study in this cohort showed that protein-energy malnutrition was associated with a higher incidence of clinical malaria ^55^. In the pooled model, sub-counties were included as fixed effect given the small number of sub-counties and the absence of regional predictors. Variables included in GLMMs are also described in Supplementary Table S1.

Environmental variables (except EVI) were scaled from 0 to 1, where 0 corresponds to the minimum observed value and 1 to the maximum. This standardization was done to obtain comparable estimates of the coefficients and to avoid convergence problems. Therefore, coefficients represent the risk ratios of malaria for a given change in meteorological variable from its minimum to its maximum value. In model outputs only coefficients for linear predictors were presented. The percent change in malaria risk between the 25th and the 50th percentile, and between the 50th and the100th percentile were presented for non-linear predictors.

Model selection was based on the Akaike Information Criterion (AIC)^56^, by considering the smallest AIC to identify the best models, as well as the difference (Δ*i*) between the AICs of each of the models and the minimum AIC found for the set of models compared. Values of Δ*i* higher than 7 indicate models that have poor fit relative to the best model, whereas values less than 2 indicate models that are equivalent to the minimum AIC model^57^.

Model fit assessment was based on DHARMa residual diagnostics for hierarchical models. A comparison between the pooled models controlling (with IRS) and not controlling (without IRS) for residual spraying was also conducted to evaluate the influence of IRS on the malaria-environment relationship. To this end, the change in the marginal effects at the mean of the environmental variables was analyzed and the percentage difference between the maximum risk for a given environmental variable in the model without IRS and the maximum risk of this variable in the model with IRS was calculated. The same comparison was made for Nagongera models with and without IRS. The analyses were performed using R Studio version 3.6.3 (https://www.r-project.org/). Marginal effects at the mean were produced with the *effect* package^58^ which allowed predictor effect plots based on 50 points covering the observed range of values of the focal predictor (i.e. a given environmental variable), while the other predictors are kept constant at their mean value^59^. The *mgcv* package^60^ was used to analyze nonlinear relationships.

### Ethical approval

Ethical approval for the cohort study was granted by the Makerere University School of Medicine Research and Ethics Committee, the Uganda National Council for Science and Technology (UNCST), the London School of Hygiene & Tropical Medicine Ethics Committee, the Durham University School of Biological and Biomedical Sciences Ethics Committee, the University of California, San Francisco Committee on Human Research and The University of Pennsylvania. Written informed consent to participate in the study was obtained from all study participants (or their designate). The cohort study was conducted according to the principles of the Declaration of Helsinki, UNCST National Guidelines for Research involving Humans as Research Participants, National and International Ethical Guidelines for Biomedical Research Involving Human as participants, the Belmont Report, and the European Convention on Human Rights and Biomedicine. The present study used secondary data from the cohort study, obtained ethical approval from the School of Public Health of the Université de Montréal Ethics Committee, and complies with to the Tri-Council Policy Statement: Ethical Conduct for Research Involving Humans.

## Supplementary Information

Supplementary Information.

## Data Availability

Cohort data are available in ClinEpiDB repository at https://clinepidb.org/ce/app/record/dataset/DS_0ad509829e . Environmental data were accessed through MODIS (https://modis.gsfc.nasa.gov/data/) and Corpernicus (https://climate.copernicus.eu/).
