# Supplementary material for: The influence of the environment and indoor residual spraying on malaria risk in a cohort of children in Uganda
Source: Sci Rep. 2022 Jul 7;12:11537. doi: 10.1038/s41598-022-15654-0 (PMC9262898; doi:10.1038/s41598-022-15654-0)

**Supplementary information**

Table S1 - Description of GLMM model variables

| Variable | Type | Description |
| --- | --- | --- |
| Sex | Factor | Female, male |
| Age at visit | Numeric | Age at each visit clinic |
| Housing type | Factor | Modern, traditional |
| Household wealth index | Factor | poorer, middle, less poor |
| Number of persons living in the house | Numeric | Number of persons living in a household |
| Meat meals per week | Numeric | Number of meat meals per week |
| Rainfall | Numeric | Cumulative rainfall averaged over 7 to 120 days (mm) |
| Minimum temperatures | Numeric | Minimum temperatures averaged over 7 to 120 days (˚C) |
| Maximum temperatures | Numeric | Maximum temperatures averaged over 7 to 120 days (˚C) |
| Humidity | Numeric | Specific humidity averaged over 7 to 120 days (kg kg^-1^) |
| Enhanced vegetation index | Numeric | Enhanced vegetation index |
| Indoor residual spraying | Factor | no spraying, phase 1, phase 2, phase3, phase 4, phase 5 |
| Additional variable for global model |  |  |
| Subcounty | Factor | Kihihi, Nagongera, Walukuba |

Table S2 – Distribution of exposure to environmental factors between 2011 - 2017

|  | **Kihihi (N=11667)** | **Nagongera (N=18071)** | **Walukuba (N=8355)** | **Overall (N=38093)** |
| --- | --- | --- | --- | --- |
| **Cumulative rainfall (mm - 20 days)** |  |  |  |  |
| Mean ± SD | 73.9 ± 43.2 | 86.0 ± 57.2 | 77.5 ± 48.1 | 80.4 ± 51.6 |
| Min - Max | 0 - 208 | 0 - 276 | 0 - 241 | 0 - 276 |
| **Minimum temperatures (C˚ - 20 days)** |  |  |  |  |
| Mean ± SD | 17.9 ± 0.550 | 18.3 ± 0.674 | 19.5 ± 0.660 | 18.5 ± 0.842 |
| Min - Max | 16.2 - 19.7 | 16.3 - 20.5 | 17.9 - 21.7 | 16.2 - 21.7 |
| **Maximum temperatures (C˚ - 20 days)** |  |  |  |  |
| Mean ± SD | 27.2 ± 1.04 | 29.0 ± 1.60 | 26.8 ± 1.23 | 28.0 ± 1.69 |
| Min - Max | 25.2 - 30.6 | 26.7 - 33.9 | 25.1 - 31.2 | 25.1 - 33.9 |
| **Humidity (kg kg-1 - 20 days)** |  |  |  |  |
| Mean ± SD | 0.0120 ± 0.00173 | 0.0134 ± 0.00200 | 0.0142 ± 0.00151 | 0.0132 ± 0.00199 |
| Min - Max | 0.00806 - 0.0147 | 0.00854 - 0.0183 | 0.0101 - 0.0176 | 0.00806 - 0.0183 |
| **Cumulative rainfall (mm - 30 days)** |  |  |  |  |
| Mean ± SD | 110 ± 58.8 | 130 ± 77.9 | 114 ± 64.2 | 120 ± 70.2 |
| Min - Max | 5.99 - 259 | 1.40 - 355 | 2.28 - 353 | 1.40 - 355 |
| **Minimum temperatures (C˚ - 30 days)** |  |  |  |  |
| Mean ± SD | 17.9 ± 0.537 | 18.3 ± 0.643 | 19.4 ± 0.637 | 18.5 ± 0.822 |
| Min - Max | 16.4 - 19.6 | 16.3 - 20.4 | 17.9 - 21.6 | 16.3 - 21.6 |
| **Maximum temperature** (**C˚ - 30 days)** |  |  |  |  |
| Mean ± SD | 27.2 ± 0.939 | 29.0 ± 1.53 | 26.8 ± 1.15 | 28.0 ± 1.63 |
| Min - Max | 25.6 - 30.1 | 27.0 - 33.2 | 25.4 - 30.7 | 25.4 - 33.2 |
| **Humidity (kg kg^-1^ - 30 days)** |  |  |  |  |
| Mean ± SD | 0.0120 ± 0.00170 | 0.0134 ± 0.00194 | 0.0142 ± 0.00149 | 0.0132 ± 0.00195 |
| Min - Max | 0.00816 - 0.0146 | 0.00880 - 0.0179 | 0.0103 - 0.0171 | 0.00816 - 0.0179 |
| **Cumulative rainfall (mm - 60 days)** |  |  |  |  |
| Mean ± SD | 217 ± 98.8 | 260 ± 129 | 229 ± 105 | 240 ± 117 |
| Min - Max | 17.5 - 450 | 10.1 - 596 | 25.8 - 591 | 10.1 - 596 |
| **Minimum temperatures (C˚ - 60 days)** |  |  |  |  |
| Mean ± SD | 17.9 ± 0.498 | 18.3 ± 0.608 | 19.4 ± 0.573 | 18.4 ± 0.797 |
| Min - Max | 16.8 - 19.4 | 16.6 - 20.2 | 18.2 - 21.4 | 16.6 - 21.4 |
| **Maximum temperatures (C˚ - 60 days)** |  |  |  |  |
| Mean ± SD | 27.2 ± 0.776 | 29.0 ± 1.35 | 26.8 ± 0.970 | 27.9 ± 1.49 |
| Min - Max | 25.9 - 29.2 | 27.4 - 32.8 | 25.5 - 29.5 | 25.5 - 32.8 |
| **Humidity (kg kg-1 - 60 days)** |  |  |  |  |
| Mean ± SD | 0.0121 ± 0.00159 | 0.0134 ± 0.00177 | 0.0141 ± 0.00140 | 0.0132 ± 0.00182 |
| Min - Max | 0.00832 - 0.0144 | 0.00961 - 0.0176 | 0.0106 - 0.0166 | 0.00832 - 0.0176 |
| **Cumulative rainfall (C˚ - 90 days)** |  |  |  |  |
| Mean ± SD | 328 ± 121 | 388 ± 159 | 344 ± 122 | 360 ± 143 |
| Min - Max | 55.3 - 593 | 79.8 - 737 | 95.8 - 723 | 55.3 - 737 |
| **Minimum temperatures (C˚ - 90 days)** |  |  |  |  |
| Mean ± SD | 17.9 ± 0.470 | 18.3 ± 0.567 | 19.4 ± 0.498 | 18.4 ± 0.766 |
| Min - Max | 16.9 - 19.3 | 17.1 - 20.0 | 18.5 - 21.1 | 16.9 - 21.1 |
| **Maximum temperatures (C˚ - 90 days)** |  |  |  |  |
| Mean ± SD | 27.2 ± 0.650 | 29.0 ± 1.15 | 26.8 ± 0.831 | 27.9 ± 1.37 |
| Min - Max | 26.1 - 28.9 | 27.4 - 32.1 | 25.7 -29.2 | 25.7 - 32.1 |
| **Humidity (kg kg^-1^ - 90 days)** |  |  |  |  |
| Mean ± SD | 0.0121 ± 0.00144 | 0.0134 ± 0.00160 | 0.0142 ± 0.00123 | 0.0132 ± 0.00167 |
| Min - Max | 0.00852 - 0.0143 | 0.00987 - 0.0172 | 0.0111 - 0.0166 | 0.00852 - 0.0172 |
| **Cumulative rainfall (mm - 120 days)** |  |  |  |  |
| Mean ± SD | 436 ± 118 | 519 ± 165 | 458 ± 118 | 480 ± 147 |
| Min - Max | 162 - 797 | 143 - 858 | 187 - 825 | 143 - 858 |
| **Minimum temperatures (C˚ - 120 days)** |  |  |  |  |
| Mean ± SD | 17.9 ± 0.429 | 18.3 ± 0.520 | 19.4 ± 0.449 | 18.4 ± 0.735 |
| Min - Max | 17.0 - 19.2 | 17.3 - 19.8 | 18.6 - 20.9 | 17.0 - 20.9 |
| **Maximum temperatures (C˚ - 120 days)** |  |  |  |  |
| Mean ± SD | 27.2 ± 0.568 | 29.0 ± 1.01 | 26.8 ± 0.741 | 27.9 ± 1.29 |
| Min - Max | 26.1 - 28.4 | 27.5 - 31.4 | 25.7 - 28.6 | 25.7 - 31.4 |
| **Humidity (kg kg^-1^ - 120 days)** |  |  |  |  |
| Mean ± SD | 0.0120 ± 0.00125 | 0.0135 ± 0.00142 | 0.0142 ± 0.00105 | 0.0132 ± 0.00153 |
| Min - Max | 0.00925 - 0.0143 | 0.0103 - 0.0168 | 0.0116 - 0.0166 | 0.00925, 0.0168 |

Table S3 – Comparative table of AICs between general models and each sub-counties for averaging periods 20, 30, 60, 90 and 120 days

| Averaging period | AIC (*Models with cubic spline)* | Difference of AIC from the smallest AIC (Δ*i)* | Nonlinear predictors in the models | AIC (models with only linear predictors) |
| --- | --- | --- | --- | --- |
| General model |  |  |  |  |
| 20 days | 27140 | 140 | Rain, humidity | 27254 |
| 30 days | 27111 | 111 | Rain, humidity | 27187 |
| 60 days | 27013 | 13 | Rain, humidity | 27115 |
| 90 days | 27000 | 0 | Rain, humidity | 27187 |
| 120 days | 27155 | 155 | Rain, humidity, maximum temperature | 27254 |
| Walukuba | | | |  |
| 20 days | 2348 | 51 | Ø |  |
| 30 days | 2342 | 45 | Ø |  |
| 60 days | 2330 | 33 | Ø |  |
| 90 days | 2314 | 17 | Ø |  |
| 120 days | 2297 | 0 | Ø |  |
| Nagongera | | | |  |
| 20 days | 13950 | 109 | Rain, humidity | 14871 |
| 30 days | 13921 | 80 | Rain, humidity | 14797 |
| 60 days | 13846 | 5 | Rain, humidity | 14754 |
| 90 days | 13841 | 0 | Rain, humidity | 14785 |
| 120 days | 13861 | 20 | Rain, humidity | 14804 |
| Kihihi | | | |  |
| 20 days | 10574 | 0 | Min temp, max temp | 10799 |
| 30 days | 10600 | 26 | Min temp, max temp | 10768 |
| 60 days | 10592 | 18 | Rain, max temp | 10743 |
| 90 days | 10614 | 40 | Rain, max temp | 10776 |
| 120 days | 10759 | 185 | Rain, humidity, max temp | 10857 |

Table S4 – Comparative table of AICs between general models with cubic splines for an averaging period 7 days, lags up to 16 weeks

| Averaging period | Lag | AIC (Models with cubic spline) | Nonlinear predictors in the models | Difference of AIC from the smallest AIC (Δ*i)* |
| --- | --- | --- | --- | --- |
| 7 days | 0 | 27719 | Humidity, maximum temperatures | 133 |
|  | 1 | 27725 | Humidity, maximum temperatures, rain | 139 |
|  | 2 | 27742 | Humidity, maximum temperatures, rain | 156 |
|  | 3 | 27698 | Humidity, rain | 112 |
|  | 4 | 27774 | Humidity, rain | 188 |
|  | 5 | 27729 | Humidity, rain | 143 |
|  | 6 | 27729 | Humidity, rain | 143 |
|  | 7 | 27725 | Humidity, rain | 139 |
|  | 8 | 27696 | Humidity, maximum temperatures, rain | 110 |
|  | 9 | 27687 | Humidity, rain | 101 |
|  | 10 | 27604 | Humidity, maximum temperatures, rain | 18 |
|  | 11 | 27670 | Humidity, maximum temperatures, rain | 84 |
|  | 12 | 27605 | Humidity, maximum temperatures | 19 |
|  | 13 | 27586 | Humidity, maximum temperatures | 0 |
|  | 14 | 27683 | Humidity, maximum temperatures | 97 |
|  | 15 | 27606 | Humidity, maximum temperatures | 20 |
|  | 16 | 27589 | Humidity, maximum temperatures | 3 |

Table S5 – Comparative table of AICs between model with cubic spline for averaging period 14 days, lags up to 16 weeks

| Averaging period | Lag | AIC (Models with cubic spline) | Nonlinear predictors in the models | Difference of AIC from the smallest AIC (Δ*i)* |
| --- | --- | --- | --- | --- |
| 14 days | 0 | 27622 | Humidity, maximum temperatures | 104 |
|  | 1 | 27717 | Humidity, rain | 199 |
|  | 2 | 27669 | Humidity, rain | 151 |
|  | 3 | 27647 | Humidity, rain | 129 |
|  | 4 | 27682 | Humidity, rain | 164 |
|  | 5 | 27617 | Humidity, rain | 99 |
|  | 6 | 27661 | Humidity, rain | 143 |
|  | 7 | 27642 | Humidity, rain | 124 |
|  | 8 | 27634 | Humidity, rain | 116 |
|  | 9 | 27611 | Humidity, rain | 93 |
|  | 10 | 27533 | Humidity, rain, maximum temperatures | 15 |
|  | 11 | 27616 | Humidity, maximum temperatures | 98 |
|  | 12 | 27563 | Humidity, maximum temperatures | 45 |
|  | 13 | 27526 | Humidity, maximum temperatures | 8 |
|  | 14 | 27591 | Humidity, maximum temperatures | 73 |
|  | 15 | 27544 | Humidity, maximum temperatures | 26 |
|  | 16 | 27518 | Humidity, maximum temperatures | 0 |

Table S6 – Percentage difference between the maximum risk of malaria in the model not controlling for IRS and the maximum risk in the model controlling for IRS

|  | Global model | | | Nagongera model | | |
| --- | --- | --- | --- | --- | --- | --- |
|  | **Maximum risk (model without IRS)** | **Maximum risk (model with IRS)** | **Difference in maximum risk between models without and with IRS (%)** | **Maximum risk (model without IRS)** | **Maximum risk (model with IRS)** | **Difference in maximum risk between models without and with IRS (%)** |
| Cumulative rainfall | 0.118 | 0.100 | -15.2 | 0.188 | 0.150 | -20.2 |
| Minimum temperature | 0.249 | 0.162 | -35.0 | 0.461 | 0.151 | -67.2 |
| Maximum temperature | 0.166 | 0.149 | -10.2 | 0.205 | 0.157 | -23.4 |
| Humidity | 0.224 | 0.141 | -37.1 | 0.286 | 0.161 | -43.7 |
| EVI | 0.166 | 0.117 | -29.5 | 0.236 | 0.144 | -39.0 |


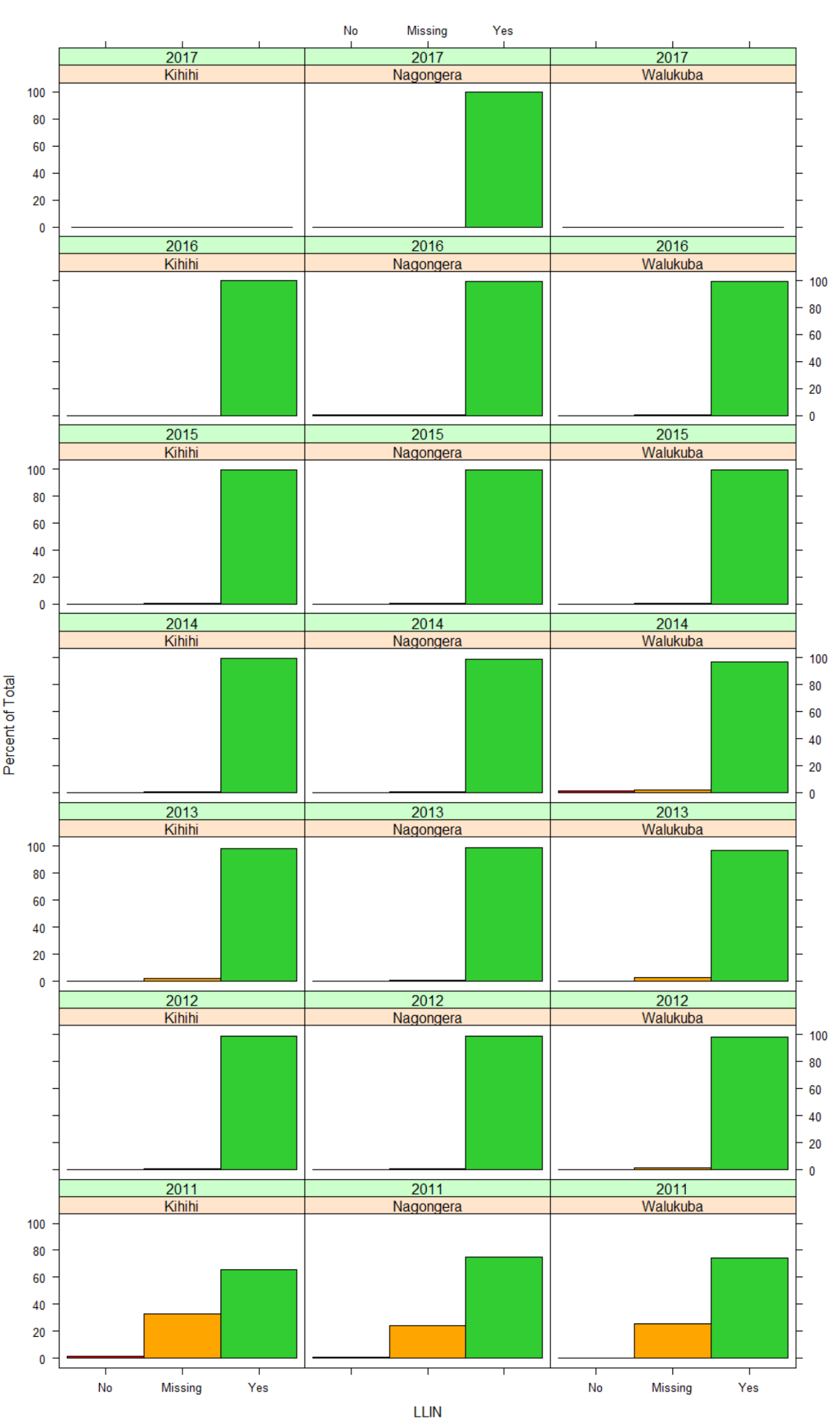
Figure S1 – Proportion of insecticide treated nets (LLIN) use per year and subcounty


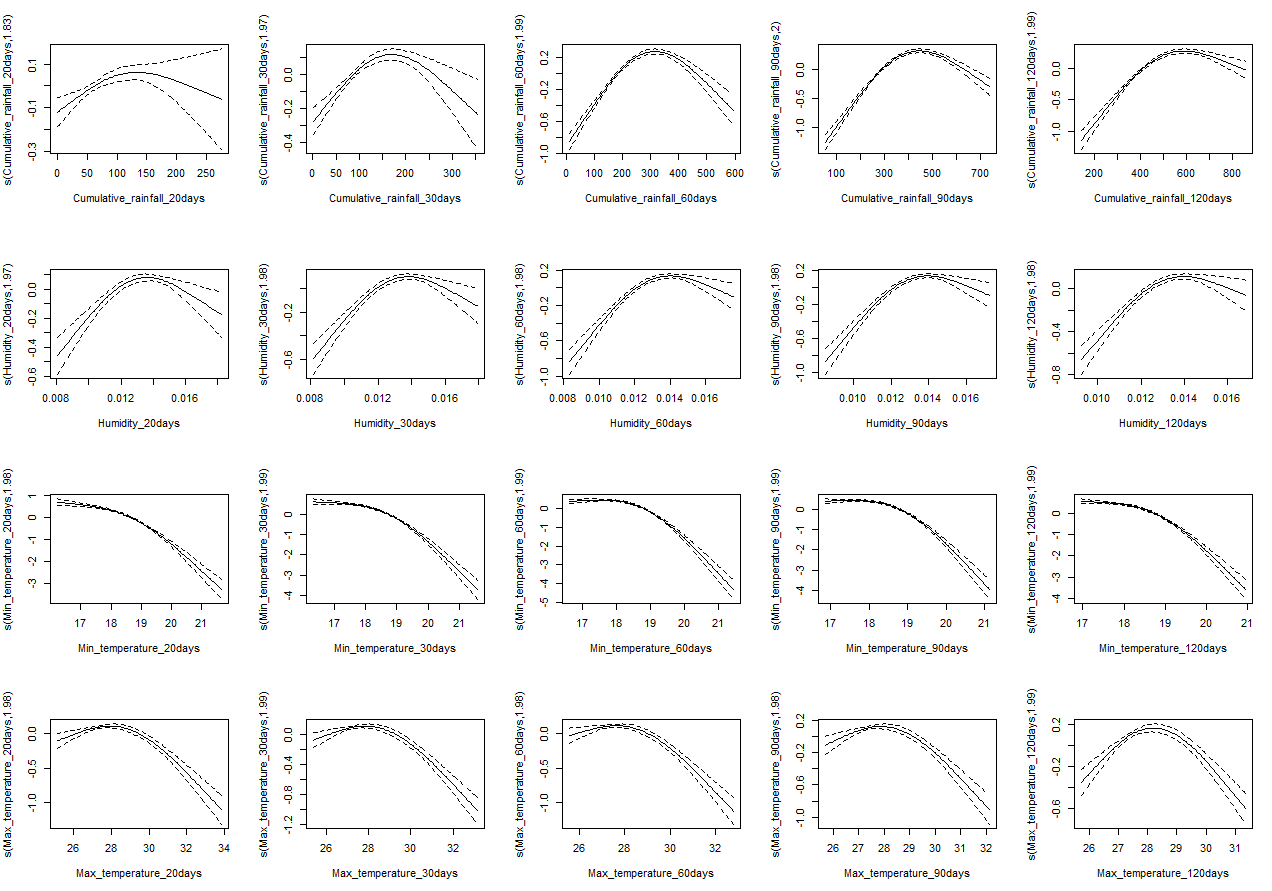
Figure S2 – Plots of bivariate analysis between malaria and cumulative rainfall, humidity, minimum temperature, and maximum temperature averaged over 20, 30, 60, 90 and 120 days for the entire cohort


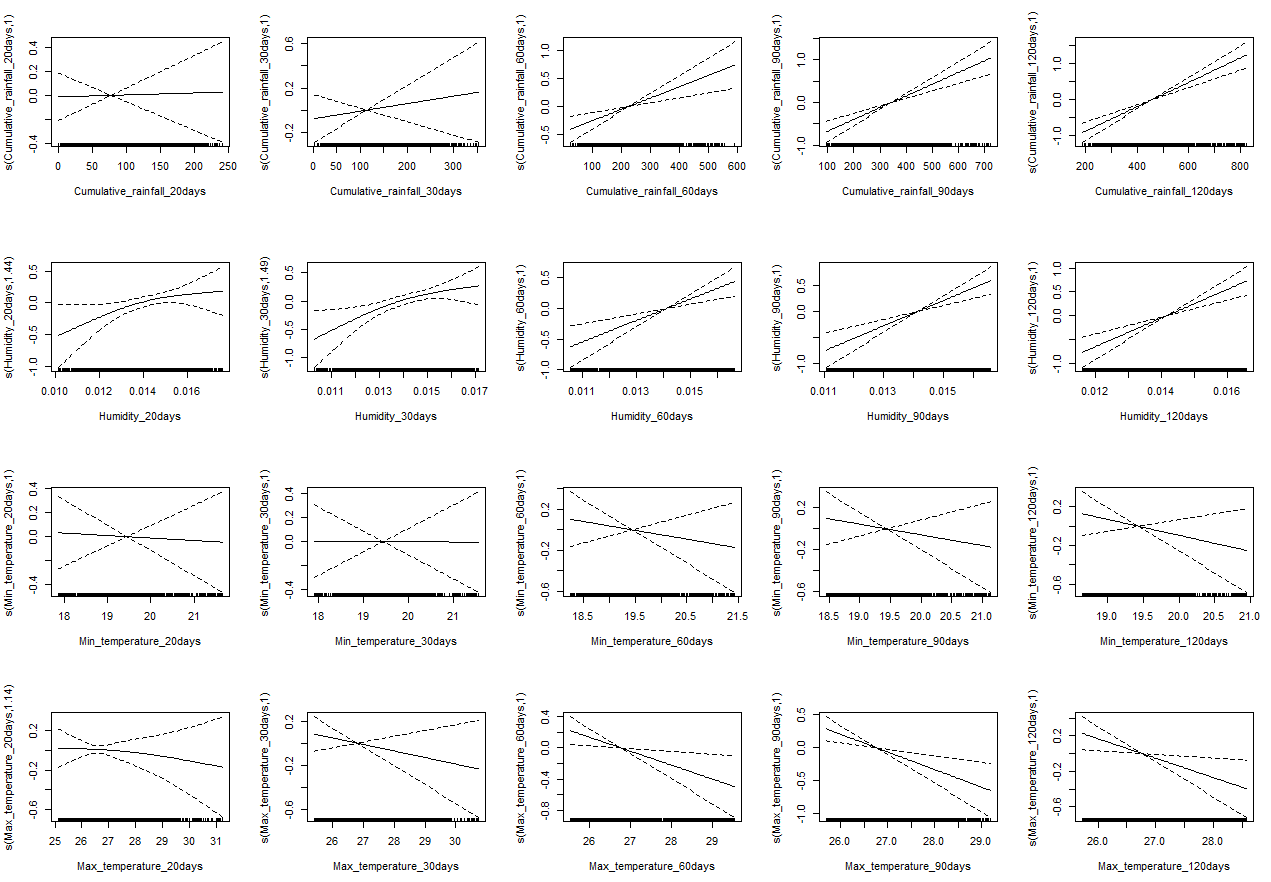
Figure S3 – Plots of bivariate analysis between malaria and cumulative rainfall, humidity, minimum temperature, and maximum temperature averaged over 20, 30, 60, 90 and 120 days for Walukuba


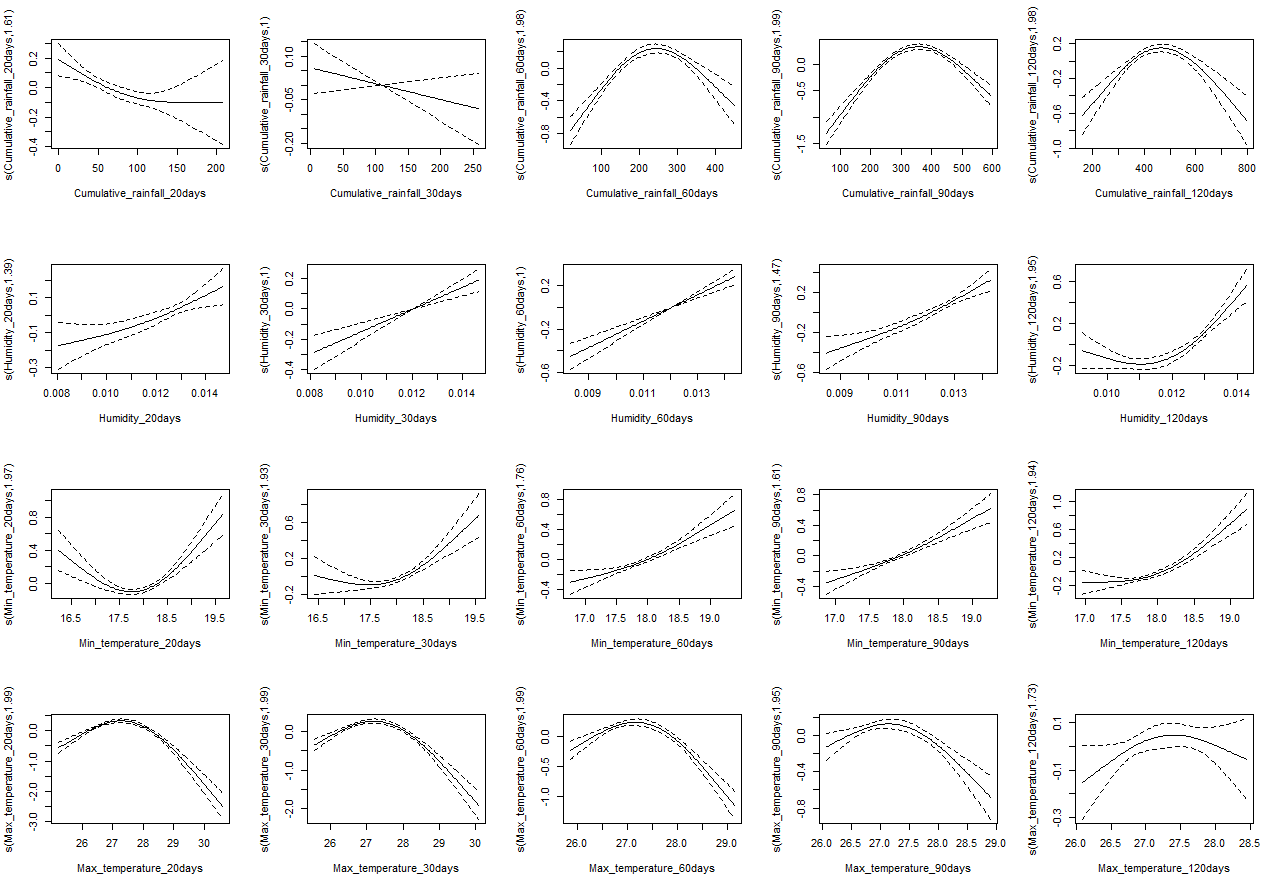
Figure S4 – Plots of bivariate analysis between malaria and cumulative rainfall, humidity, minimum temperature, and maximum temperature averaged over 20, 30, 60, 90 and 120 days for Kihihi


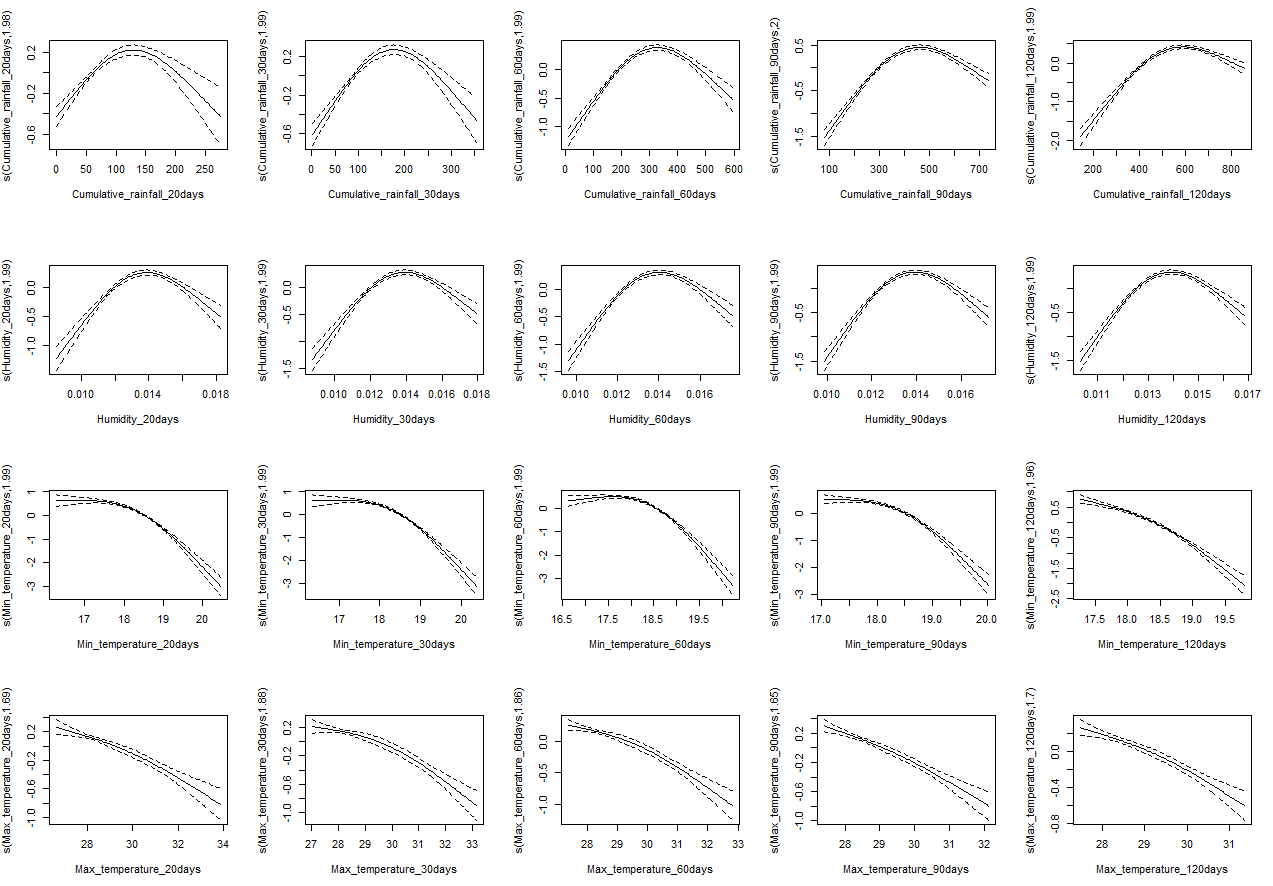
Figure S5 – Plots of bivariate analysis between malaria and cumulative rainfall, humidity, minimum temperature, and maximum temperature averaged over 20, 30, 60, 90 and 120 days for Nagongera


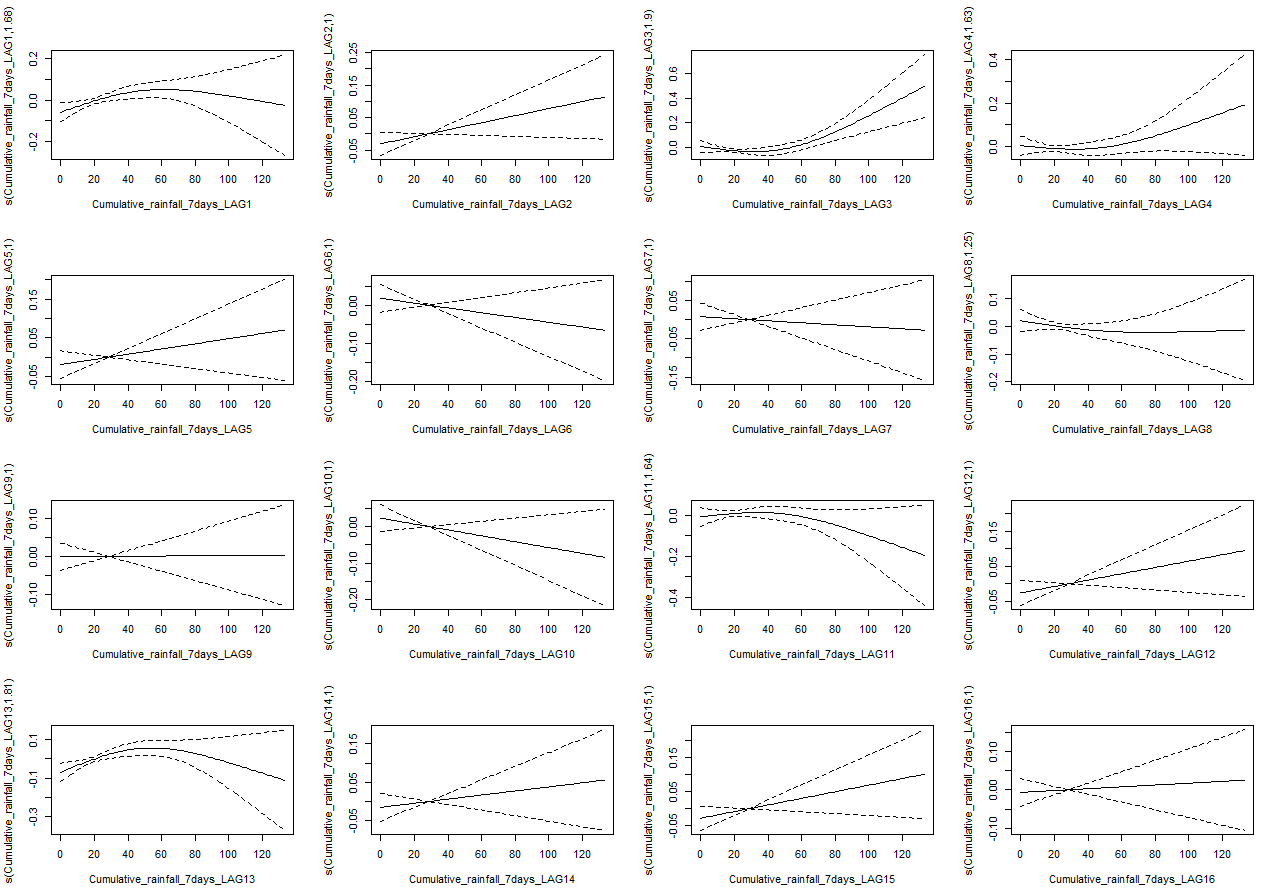
Figure S6 – Plots of bivariate analysis between malaria and cumulative rainfall averaged over 7 days, lags up to 16 weeks, for the entire cohort


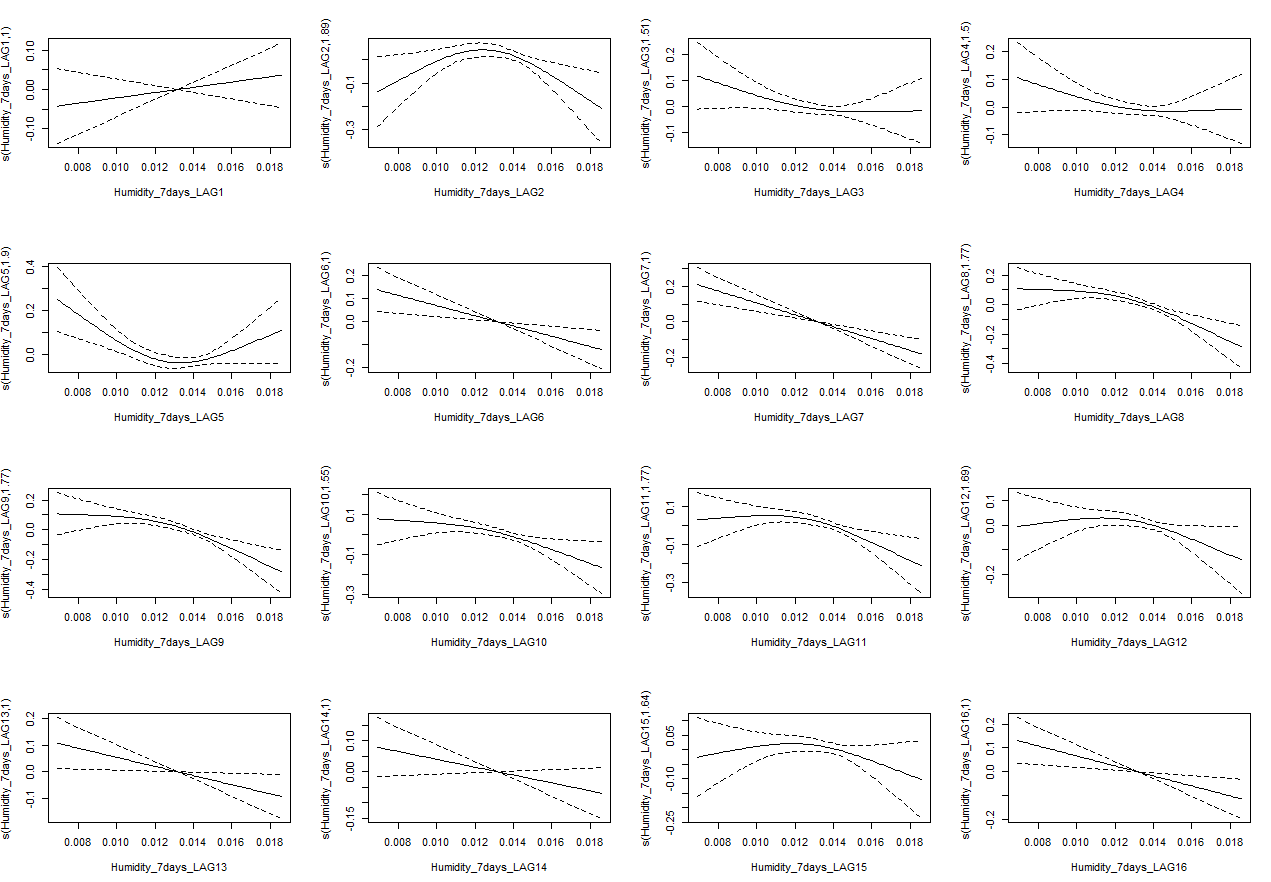
Figure S7 – Plots of bivariate analysis between malaria and humidity averaged over 7 days, lags up to 16 weeks, for the entire cohort


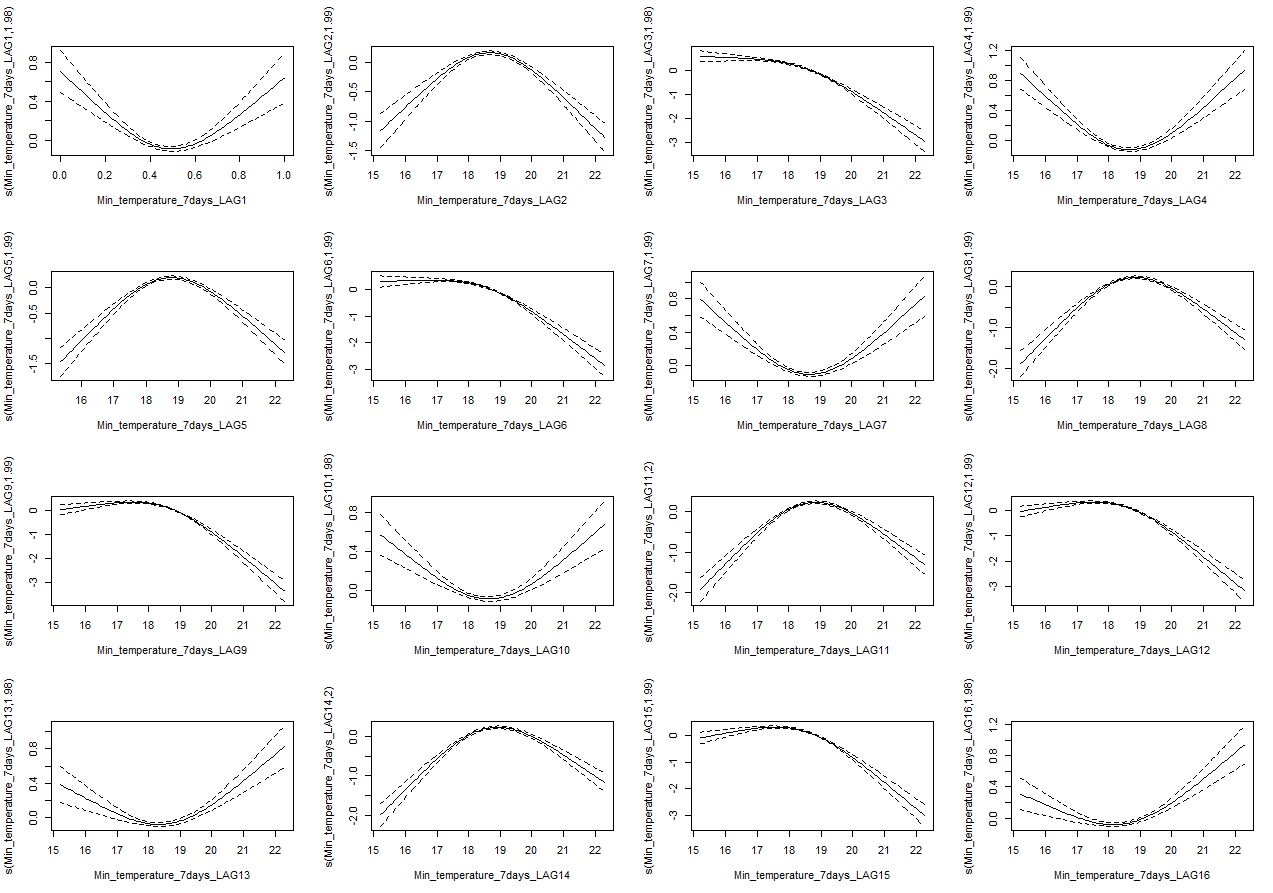
Figure S8 – Plots of bivariate analysis between malaria and minimum temperature averaged over 7 days, lags up to 16 weeks, for the entire cohort


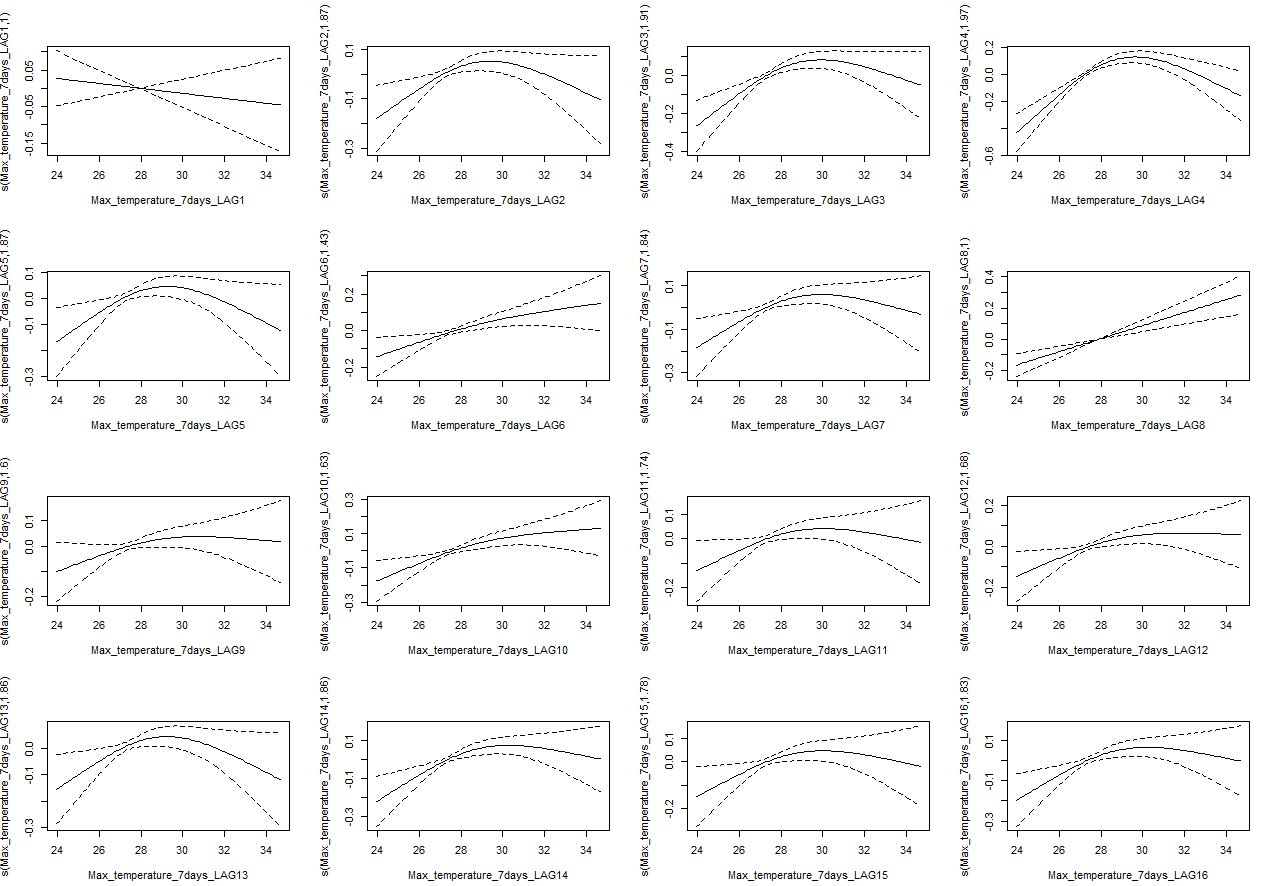
Figure S9 – Plots of bivariate analysis between malaria and maximum temperature averaged over 7 days, lags up to 16 weeks, for the entire cohort

Figure S10 - Global model (with IRS) diagnosis


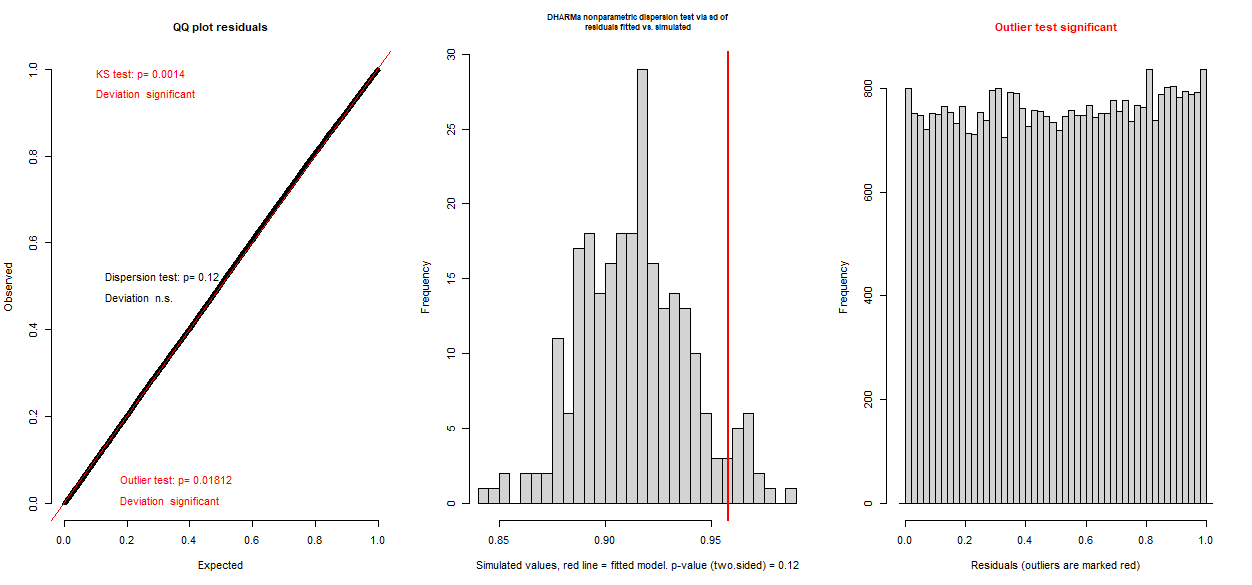


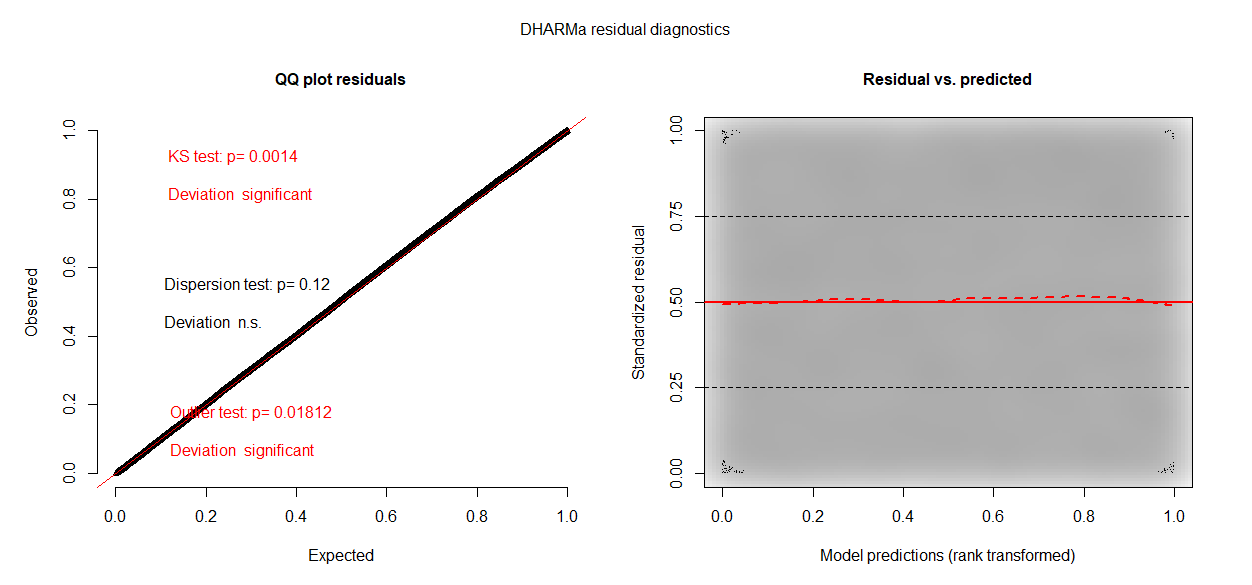


Figure S11 - Kihihi model diagnosis


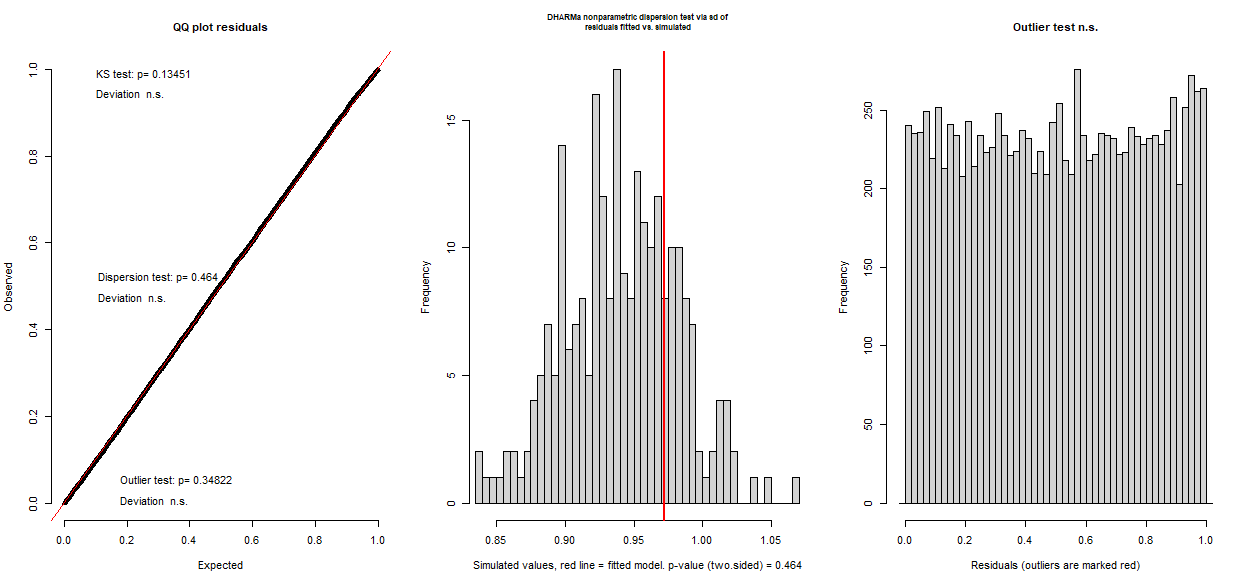


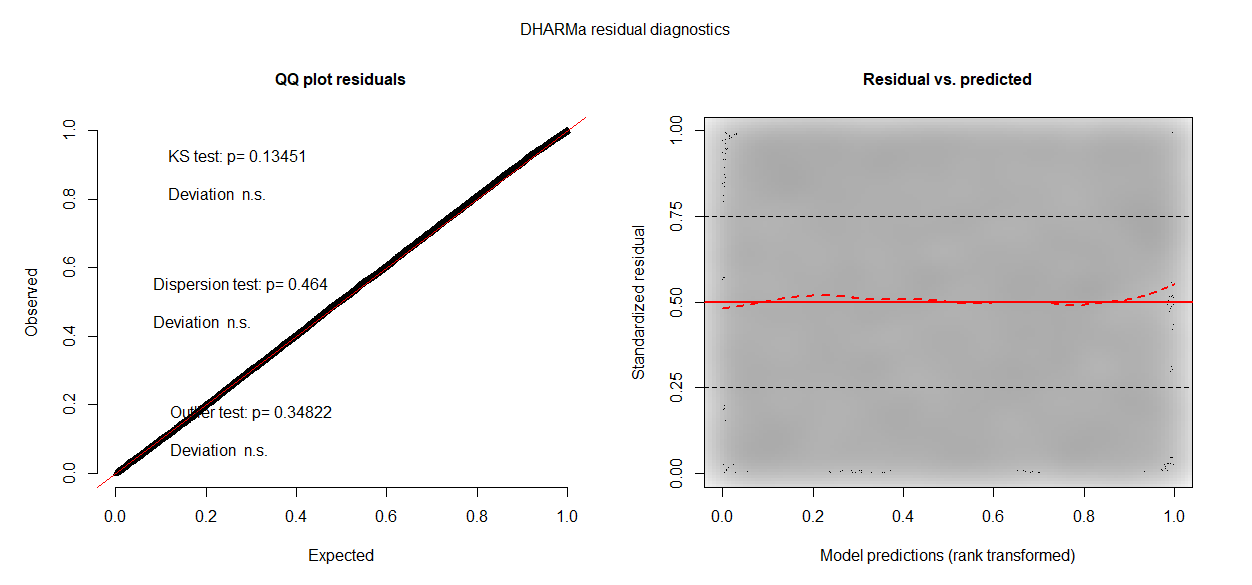


Figure S12 – Walukuba model diagnosis


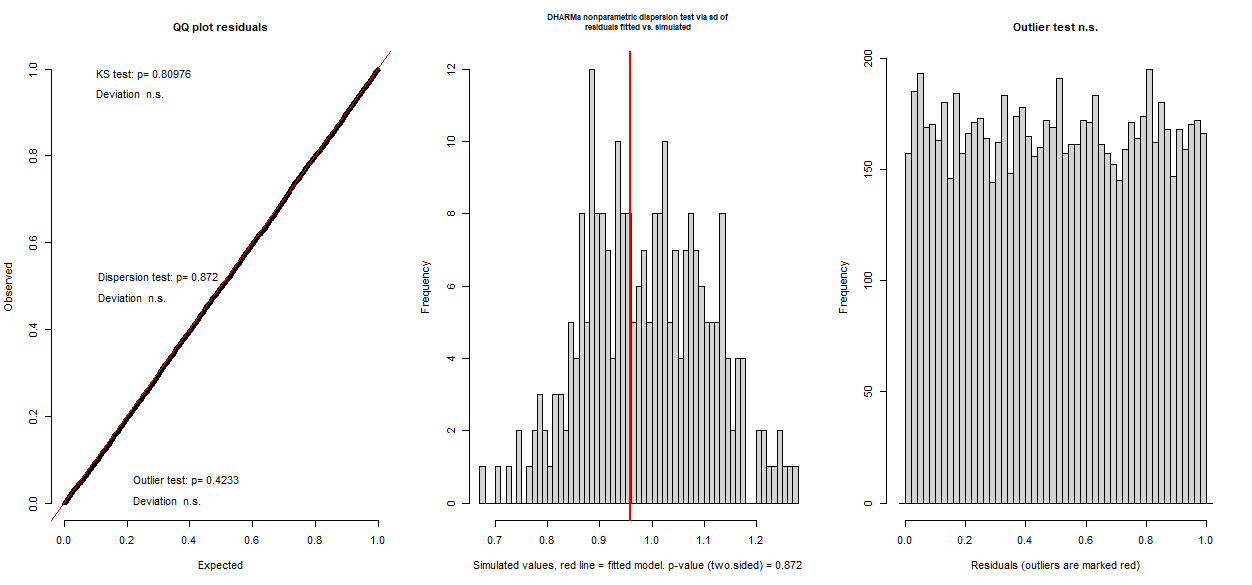


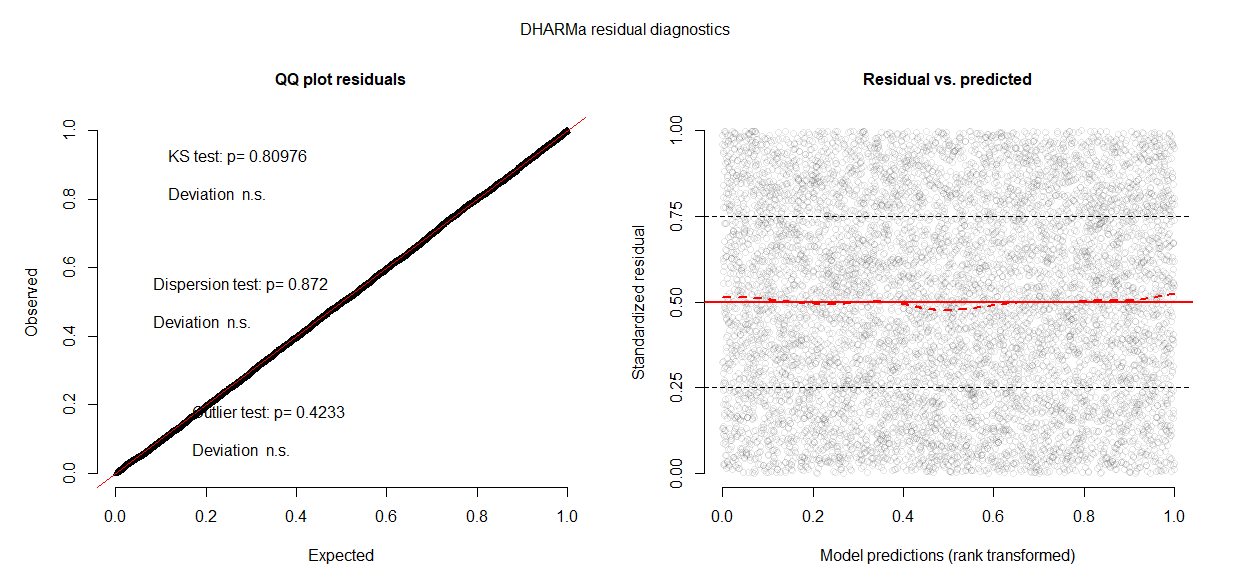


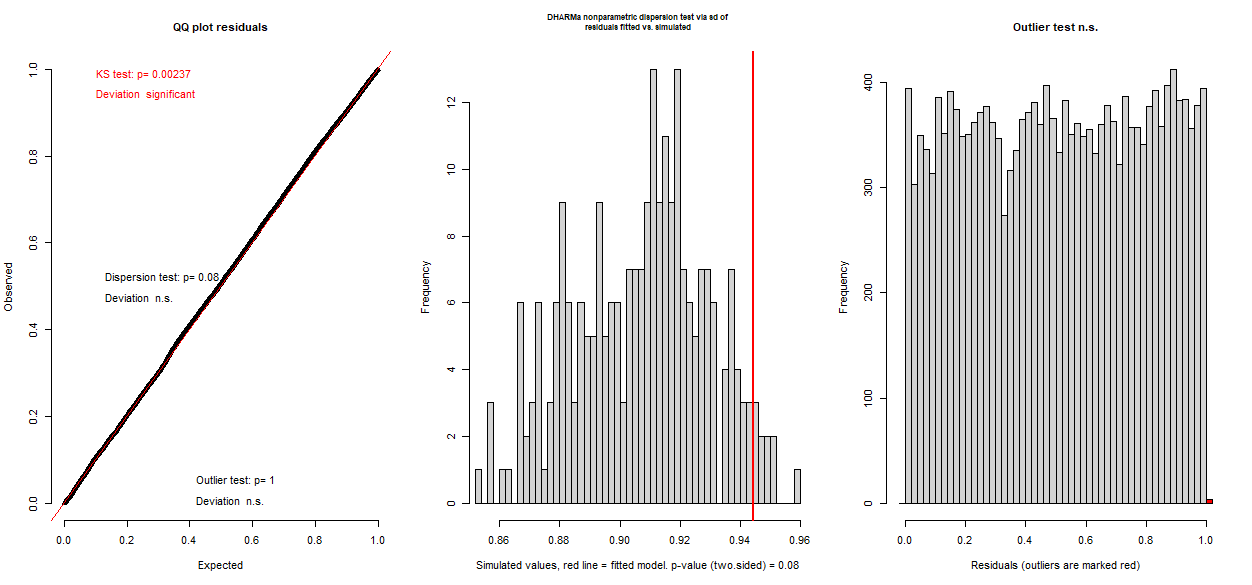
Figure S13 – Nagongera model (with IRS) diagnosis


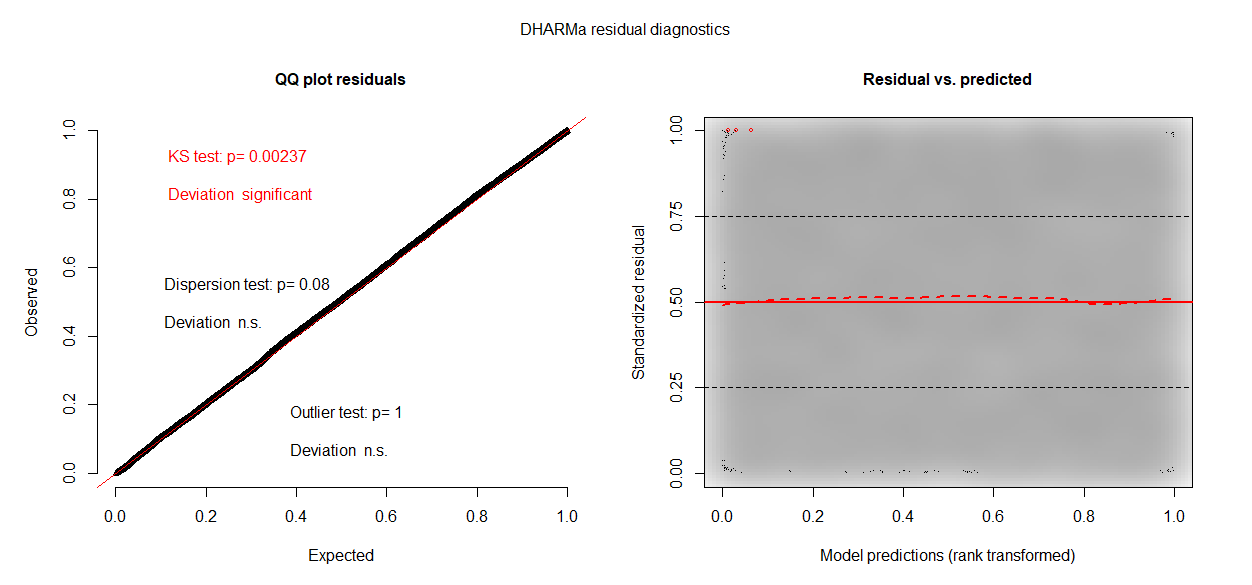

Supplement: Supplementary file 1 — Supplementary Information. [file 41598_2022_15654_MOESM1_ESM.docx]
